# A Dutch nationwide pediatric cardiac arrest registry with long-term follow-up – towards an international prognostication guideline

**DOI:** 10.1016/j.resplu.2025.100976

**Published:** 2025-05-09

**Authors:** Marijn Albrecht, Maayke Hunfeld, Annemieke Arkesteijn-Muit, Karolijn Dulfer, Matthijs de Hoog, Gabry de Jong, Rogier de Jonge, Aldert Lamoré, Vinay Nadkarni, Corinne Buysse, PROGNOSE Group Collaborative Investigators, Nikki Schoenmaker, Annelies van Zwol, Geanne Krabben-de Vlaam, Nicole de la Haye, Jennifer Walker

**Affiliations:** f Amsterdam UMC, Department of Pediatric Intensive Care, University of Amsterdam, Amsterdam, the Netherlands; g Radboud University Medical Center, Department of Pediatric Intensive Care Medicine, Nijmegen, the Netherlands; h UMCG Beatrix Children’s Hospital, Department of Pediatric Intensive Care, Groningen, the Netherlands; i Maastricht UMC+ MosaKids Children’s Hospital, Department of Pediatric Intensive Care, Maastricht, the Netherlands; j UMCU Wilhelmina Children’s Hospital, Department of Pediatric Intensive Care, Utrecht, the Netherlands; a Department of Neonatal and Pediatric Intensive Care, Division of Pediatric Intensive Care, Erasmus MC Sophia Children’s Hospital, Rotterdam, the Netherlands; b Department of Pediatric Neurology, Erasmus MC Sophia Children’s Hospital, Rotterdam, the Netherlands; c Department of Child and Adolescent Psychiatry/Psychology, Erasmus MC Sophia Children’s Hospital, Rotterdam, the Netherlands; d Department of Information Technology, Erasmus MC, Rotterdam, the Netherlands; e Department of Anesthesiology and Critical Care Medicine, The Children’s Hospital of Philadelphia, University of Pennsylvania, Philadelphia, PA, United States

**Keywords:** Pediatric cardiac arrest, Registry, post-ROC protocol, Prognostication

## Abstract

**Aims:**

Pediatric cardiac arrest is associated with high mortality and significant morbidity among survivors. International guidelines for prognostication remain limited due to small heterogeneous patient populations, variable post-return of circulation diagnostics, and insufficient long-term follow-up. Pediatric Resuscitation Prognostication and Outcomes Registry (PROGNOSE) is a Dutch nationwide, multicenter registry aiming to standardize data collection, establish uniform neuromonitoring reporting, and implement structured follow-up protocols.

**Methods:**

The Pediatric Resuscitation Prognostication and Outcomes Registry (ClinicalTrials.gov ID: NCT06938009) collects data on pediatric cardiac arrest across Dutch pediatric intensive care units, extending the pediRES-Q collaborative. It includes patients <18 years with out-of-hospital cardiac arrest requiring emergency services and in-hospital cardiac arrest patients admitted to academic hospitals. Return of circulation is defined as sustained spontaneous circulation or via extracorporeal support. Exclusions include pre-existing Do Not Resuscitate orders or neonates < 24 h. The registry captures pre-hospital factors, resuscitation characteristics, post-return of circulation care, neuroprognostication markers (biomarkers, electroencephalography, imaging), and long-term outcomes. Structured follow-up occurs at 3–6 months, 12 months, and evaluations through age 17 for neurodevelopmental, psychosocial, and functional outcomes.

**Conclusion:**

The Pediatric Resuscitation Prognostication and Outcomes Registry (PROGNOSE) represents the first nationwide initiative to standardize data collection on pediatric cardiac arrest, post-return of circulation care and implement structured follow-up protocols in the Netherlands. This registry aims to address critical knowledge gaps, providing foundation for evidence-based prognostication, clinical decision-making, and long-term care policy recommendations. Future expansion efforts will focus on integrating pre-hospital data, extending follow-up into young adulthood, and strengthening international collaboration through the pediRES-Q network.

## Introduction

Limited evidence-based international guidance exists for prognostication in pediatric cardiac arrest, despite high mortality and morbidity rates among survivors. The annual incidence of pediatric out-of-hospital cardiac arrest (pOHCA) is 9.0–19.7 per 100,000 person-years, with > 7,000 cases in the United States and 350 in the Netherlands.^1^, ^2^, ^3^, ^4^, ^5^ Survival remains low at 8–10%, particularly in infants.^1^, ^2^, ^3^, ^4^, ^5^ Pediatric in-hospital cardiac arrest (pIHCA), affecting 1.4% of PICU admissions and about 15,000 U.S. children annually, has better outcomes (10–65%) due to advancements in rapid response systems, cardiopulmonary resuscitation (CPR) quality, and post-return of circulation (ROC) care.^6^, ^7^, ^8^, ^9^, ^10^

In patients with ROC, initial ischemic damage and reperfusion injury may lead to hypoxic ischemic brain injury (HIBI), causing a spectrum of physical, cognitive, neurological, and social-emotional impairments.^11^ These effect psychological well-being, quality of life, and family dynamics, both short- and long-term.^12^, ^13^ Survivors often face reduced school participation, increased healthcare costs, and challenges in managing daily life and engaging in societal activities during (young) adulthood.^12^, ^14^, ^15^ As such, the impact extends beyond the individual, carrying the potential for family and societal consequences.

Accurate prognostication is crucial to guide clinical decision-making, plan long-term care, and estimate the broader societal impact of pediatric cardiac arrest, including implications for education, employment, and healthcare needs. It prevents false pessimism in withdrawing life-sustaining therapy (WLST) and false optimism that may lead to prolonged suffering and resource strain. The lack of strong evidence-based international guidance stems from small heterogeneous patient samples, inconsistent post-ROC diagnostics, uncertain death causes and timing, and limited long-term follow-up with variable intervals and crude outcome measurements.^10^, ^16^, ^17^, ^18^, ^19^ Prognostication remains especially challenging in comatose children with preserved brainstem reflexes, requiring a multimodal approach: observation beyond 72 h repeated neurological exams, early continuous electroencephalography (EEG), brain Magnetic Resonance Imaging (MRI) (days 3–5), lactate levels and certain cerebral biomarkers.^10^, ^17^

Long-term follow-up into adulthood, including the transition to young adulthood, is essential for detecting late-emerging deficits, guiding care, and supporting development. However, few pediatric intensive care units (PICUs) offer structured multidisciplinary programs, which require a robust healthcare system, accessible clinics, and insurance support.^12^, ^20^, ^21^ The Netherlands provides an exemplar model for follow-up based on already existing national follow-up protocols for PICU survivors.^22^

To advance this field, nationwide collaboration is essential through standardized reporting and data collection on multimodal prognostication investigations and long-term follow-up of pediatric cardiac arrest (CA) survivors. This raises the primary research question: how can standardized data collection on post-ROC care, uniform reporting of neuromonitoring findings, and structured follow-up protocols improve prognostication? As a key step toward this goal, we aim to describe the development of a Dutch nationwide pediatric cardiac arrest registry, facilitating coordinated IHCA and OHCA data collection, post-ROC diagnostics, and structured follow-up after hospital discharge.

## Methods

### Foundation of the study

The Pediatric Resuscitation Prognostication and Outcomes Registry “PROGNOSE” builds on over two decades of pediatric resuscitation data collection at the Erasmus MC Sophia Children’s Hospital, one of seven PICUs in the Netherlands.

In 2012, a multidisciplinary follow-up clinic was established to monitor long-term outcomes in pediatric cardiac arrest survivors through semi-structured interviews, physical and neurological examinations, and neuropsychological testing.^12^ It contributed to the 2016 development of a national guideline for post-PICU care.^21^, ^22^ In the Netherlands, follow-up is covered by mandatory health insurance and embedded in the standard healthcare system, ensuring universal access and high attendance due to short travel distances.

Since 2015, Erasmus MC Sophia Children’s Hospital has been part of the pediRES-Q network, a global network of over 60 PICUs focused on improving pediatric resuscitation outcomes, which has strengthened our data infrastructure and supported multicenter collaboration.^10^, ^23^, ^24^, ^25^, ^26^, ^27^, ^28^ PediRES-Q has received funding and in-kind contribution for the data coordinating center from the Laerdal Foundation, Zoll Medical, Philips Medical, the American Heart Association, and the Children’s Hospital of Philadelphia Endowed Chair for Critical Care Medicine funds. In the Netherlands, we leveraged the SICK (“Sectie Intensive Care Kinderen”, part of the Dutch Society for Pediatrics) research network to establish a national pediRES-Q Hub, focused on post-ROC care and long-term outcomes, aligned with international and national guidelines.^10^, ^21^, ^22^, ^24^, ^29^ Funding from ZOLL Medical, the foundation “Stichting Vermeer14”, and a parent sponsor, ensures sustainability, with participating centers receiving compensation for study initiation and data entry.

### Study protocol and population

PROGNOSE is a multicenter, prospective longitudinal registry capturing pediatric IHCA and OHCA events across the Netherlands. Designed as a quality improvement initiative, the registry provides hypothesis-generating research on post-ROC care, prognostication, and long-term outcomes. Launched on June 1, 2023, data collection is ongoing and evolves with new insights in the field. The registry was initiated at the Erasmus MC and gradually expanded to include all Dutch academic pediatric hospitals: Erasmus MC Sophia Children’s Hospital, Amsterdam UMC Emma Children’s Hospital, UMCU Wilhelmina Children’s Hospital, Radboud university medical center Amalia Children’s Hospital, Maastricht UMC + MosaKids Children’s Hospital, UMCG Beatrix Children’s Hospital, and LUMC Willem-Alexander Children's Hospital.

The protocol (version 2.0 dd 01–02-2024) was developed by the coordinating team at Erasmus MC Sophia Children’s Hospital in collaboration with all participating centers. Oversight is provided by the SICK Steering Committee. The registry is conducted in compliance with national legislation, Good Clinical Practice guidelines, and the Declaration of Helsinki.^30^ Data processing complies with EU General Data Protection Regulation (GDPR) and the Dutch Implementation Act of the GDPR (in Dutch: Algemene Verordening Gegevensbescherming en Uitvoeringswet Algemene Verordening Gegevensbescherming). Approval was granted by the Erasmus MC Medical Ethical Committee (MEC-2023–0133), and the study is registered at ClinicalTrials.gov (ID: NCT06938009).^31^ Site investigators are responsible for data collection, and the Erasmus MC team coordinates data governance, ethics, and dissemination.

Eligible patients are under 18 years of age who experience OHCA requiring EMS involvement, or IHCA and are either already admitted or subsequently transferred to a participating academic pediatric hospital. CA is defined as pulselessness with ≥ 1 min of chest compressions. CPR follows European Resuscitation Council pediatric guidelines.^19^ ROC is defined as sustained spontaneous circulation or extracorporeal support. Exclusion criteria include a pre-existing Do Not Resuscitation order, or neonates < 24 h (typically due to perinatal asphyxia).

Children who do not achieve ROC at the scene in OHCA cases are not yet included. However, those brought to an academic ED without ROC are included, though systematic inclusion of cases from non-academic EDs has not yet been implemented.

### Data collection

Data are abstracted from electronic medical records, encompassing all routinely documented clinical information. The registry uses Castor, a secure electronic data capture system (Castor, Amsterdam, The Netherlands; https://www.castoredc.com/electronic-data-capture-system/). Data is pseudonymized, and the key to the code is safeguarded by the principal investigator at each site. Inter-center data sharing is anonymous.

**Fig. 1:**
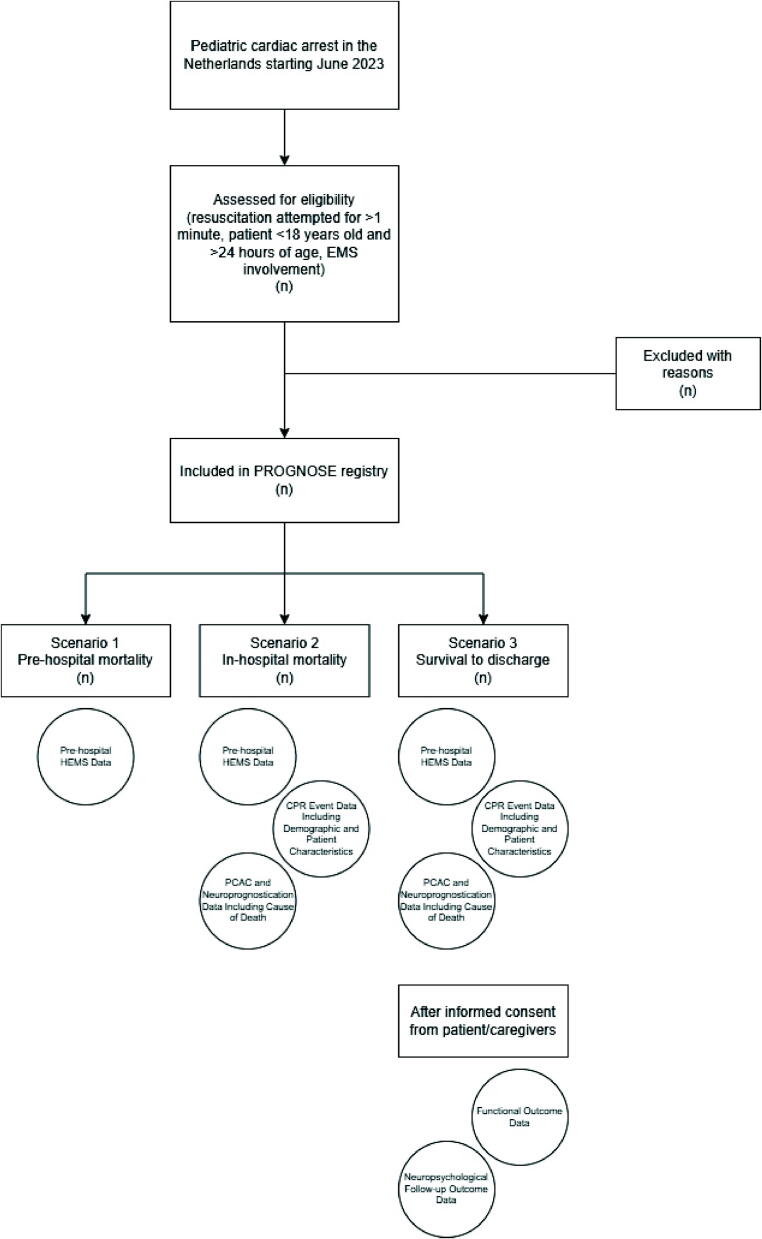
**Overview of ‘PROGNOSE’ inclusion.** Abbreviations: EMS = Emergency Medical Services, HEMS = Helicopter Emergency Medical Services, CPR = Cardiopulmonary Resuscitation, PCAC = Post-Cardiac Arrest Care.

Variables are collected across five domains (**appended list of variables**): pre-hospital factors, CPR characteristics, post-ROC care and prognostication metrics, and long-term follow-up outcomes (Fig. 1). The database structure and variable definitions follow the pediRES-Q network, promoting international standardization. The full pediRES-Q variable template is available at: https://www.pedires-q.org/document-library. As a quality improvement initiative, data collection will continue indefinitely and adapts with emerging insights.

Given the multicenter and international collaboration embedded in the registry’s design, data sharing requires careful navigation of regulatory frameworks. Within the Netherlands, data use is governed by the SICK Steering Committee. For transfers outside the European Economic Area (EEA), GDPR-compliant protections—including Standard Contractual Clauses (SCCs), encryption, and pseudonymization—are strictly applied. These measures ensure that international collaboration can proceed securely, while upholding ethical standards and protecting patient confidentiality.

### Objectives

The primary objective is to evaluate post-ROC care, neuroprognostication strategies and long-term outcomes for pediatric CA survivors admitted to Dutch PICUs. The registry supports the implementation of a standardized post-ROC protocol and provides a structured platform for longitudinal follow-up.

#### Post-ROC care and neuromonitoring (Supplemental Table 1 and 2**and appended list of variables**)

Based on the 2019 Topjian scientific statement,^10^ the registry collects data on include respiratory, hemodynamic, metabolic and temperature control. High-resolution monitoring data (e.g., ventilation, oxygenation, blood pressure) may be submitted when available. Neurological monitoring is multimodal. Structured neurological exams at defined time points include assessments of brainstem reflexes, motor responses, and consciousness. Seizure events, epilepsy diagnoses, and antiepileptic treatment initiation are recorded. Continuous EEG, when available, captures seizure activity, and background suppression; otherwise, amplitude-integrated EEG is used. CT and MRI findings-especially diffusion-weighted imaging and ADC mapping-are documented to assess hypoxic-ischemic injury. Somatosensory evoked potentials (SSEP) and neuroprognostic biomarkers (e.g. NSE, S100B and MBP, NFL) are included. Prognostication is discouraged before 72 h post-ROC, due to confounding effects of sedation, hypothermia, and metabolic disturbances.^10^ If seizures occur, antiepileptic therapy is initiated in consultation with pediatric neurology, avoiding excessive neuromuscular blockade to preserve clinical assessment.

#### Follow-up protocol for survivors (Supplemental Table 3 and 4)

The Dutch national post-PICU follow-up guideline provides a framework for standardized care and long-term outcome tracking.^22^ Initial assessment occur 3–6 months post-discharge by a pediatric intensivist, neurologist and psychologist, covering neurodevelopment, physical and emotional health, and quality of life. Screening includes post-traumatic stress symptoms in patients and caregivers, behavioral and neuropsychological testing for at-risk children, and physical recovery measures. Psychosocial support is integral to this phase.

Follow-up is repeated at 12 months for persistent issues (symptoms, developmental concerns or behavioural problems) and includes referrals to subspecialists if needed. Long-term follow-up at 5–6 years, 11–12 years, and 15–17 years ensures that late-onset deficits are detected, particularly as academic and social demands increase, with evaluations focusing on cognitive, motor, and functional health to support full participation in daily life. However, longer-term follow-up is not available at every PICU hospital in the Netherlands. Given the emotional burden on families, informed consent for long-term data collection is deferred to follow-up visits rather than the acute ICU phase, ensuring ethical and feasible participation in research and clinical monitoring. In accordance with approval from the Medical Ethical Committee, informed consent for ICU and prehospital data collection was waived due to the minimal risk nature of the registry and the acute circumstances; data from patients who died before or during hospital admission are therefore included without requiring consent.

#### Research questions and outcomes

The following research questions will be addressed:

• What is the diagnostic value of neurological exams, EEG, neuroimaging, and biomarkers in the post-ROC phase?
• How do survivors fare in terms of neurological (PCPC, POPC, FSS) and neuropsychological outcomes over time?

What are the health status, quality of life, and psychosocial impacts on families? As the registry expands and systematic inclusion of prehospital data improves, future research questions will include epidemiological outcomes such as:

• The incidence of pediatric CA (both in-hospital and out-of-hospital) in the Netherlands.

The incidence of ROC, causes of death in the PICU, and survival to hospital discharge. The primary objective of this registry was to implement a standardized post-ROC protocol that promotes and enhances optimal care, neuromonitoring, and structured long-term follow-up for pediatric cardiac arrest survivors and their families/caregivers. By predicting long-term neuropsychological outcomes, the registry aims to facilitate timely interventions to help children thrive and support caregivers.

Beyond its immediate clinical applications, the registry will serve as a platform for needs analyses and future hypothesis-driven research, with long-term goals to:

• Improve post-ROC outcomes by refining prognostication, identifying risk factors for poor recovery and facilitators of good recovery, and developing therapeutic interventions.
• Reduce the societal impact of pediatric cardiac arrest by enhancing participation in education and employment, lowering healthcare costs, and improving overall quality of life.

To maximize the impact of this research, findings from the registry will be submitted for open-access analysis and publication in peer-reviewed journals and shared with guideline organizations such as the Dutch Resuscitation Council, European Resuscitation Council, and International Liaison Committee on Resuscitation to inform clinical implementation and guideline development.

### Statistical analysis

As a prospective quality improvement registry, PROGNOSE does not have a predefined sample size calculation. Inclusion is continuous and based on all eligible pediatric cardiac arrest cases across participating centers. Based on historical data and current participation rates, we anticipate enrolling approximately 100–150 patients per year. The registry is designed as a prospective data-collection on which research questions can be applied in retrospect, and all centers are encouraged to propose relevant questions. Descriptive analyses will be performed to summarize baseline characteristics, resuscitation details, and follow-up outcomes. Depending on sample size and data completeness, multivariable logistic, linear regression or longitudinal mixed models may be used to explore associations between early clinical variables, neuroprognostic markers, and long-term outcomes. Analyses will be exploratory and hypothesis-generating. Missing data will be assessed, and appropriate methods such as multiple imputation may be applied when feasible. In the long term, PROGNOSE aims to support the development of predictive models to identify modifiable and non-modifiable risk factors associated with outcomes, using multimodal clinical, physiological, and biomarker data to guide individualized post-cardiac arrest care and neuroprognostication.

## Discussion

The PROGNOSE registry, launched in June 2023, aimed to standardize post-ROC care, optimize neuromonitoring and long-term follow-up, and improve prognostication and post-arrest outcomes for pediatric cardiac arrest survivors. By predicting long-term neuropsychological outcomes, the registry facilitates timely interventions in hopes of helping children achieve an independent and fulfilling life, while also supporting caregiver well-being. Additionally, it seeks to reduce the societal impact of pediatric cardiac arrest by improving participation in education and employment.

As a pediRES-Q National Hub, the registry enables systematic data collection, serving as a foundation for hypothesis-driven research and the development of an evidence-based prognostication guideline. Currently, six out of seven Dutch PICUs are actively participating, with over 150 cases included within the first 1.5 years. The seventh center has recently joined and is in the process of starting data collection, but has not yet contributed inclusions. While early feasibility data demonstrate successful multicenter collaboration (overall data completeness for ICU variables of 80%)—enabled by a core set of standardized variables (with 100% data completeness)—ongoing efforts aim to expand prehospital and non-academic ED data inclusion to improve data completeness, support national epidemiological analyses, and ensure long-term sustainability.

### Key challenges and opportunities for expansion and improvement

There are several key challenges currently faced by the registry, and addressing these challenges presents several opportunities for registry expansion and improved patient care.

A key challenge identified through preliminary insights is the variability in post-ROC care and neuromonitoring across participating PICUs. Despite a standardized protocol, the full bundle—particularly EEG and MRI for prognostication—is not consistently applied due to limited 24/7 EEG availability, logistical challenges with MRI in critically ill children, and differences in institutional protocols. Addressing this variability presents an opportunity to strengthen collaboration through expanding EEG and MRI availability and improve adherence to shared protocols.

Currently, PROGNOSE does not include OHCA cases where children die at the scene without achieving ROC, which introduces selection bias and limits epidemiological analysis. Data from the Erasmus MC, which serves 25% of the Dutch population, suggests that 25% of HEMS-attended OHCA cases fall into this category.^32^ Expanding the registry to include these non-survivors, in collaboration with Dutch HEMS teams, would reduce bias and enable a more accurate understanding of pediatric OHCA incidence, survival disparities, and opportunities for community-based intervention. This expansion could also support the development of nationwide initiatives to improve CPR training, AED accessibility, and community-based interventions, particularly in low-SES regions where bystander response rates are lower.

Although our center provides structured follow-up through adolescence, many PICUs lack long-term monitoring beyond one year. Many survivors receive little to no medical follow-up after turning 18. This creates a gap in transitional care, particularly for young adult survivors at risk for unrecognized cognitive or psychosocial impairments that can impact education, employment, and social participation. Establishing centralized long-term follow-up and structured transition-of-care models would help identify impairments earlier and support full reintegration into education, work, and society. This could inform the development of national rehabilitation pathways and targeted support programs.

Finally, ensuring sustainability and policy integration will be essential for the registry’s long-term impact. Although initial funding from ZOLL Medical, the foundation “Stichting Vermeer14”, and a parent sponsor has enabled PROGNOSE’s launch, long-term financial support remains uncertain. Ensuring continued funding and reducing data collection burden are key priorities. Automating data collection through integration with electronic medical records (EMRs) would reduce workload for participating centers and improve data completeness. Furthermore, advocating for mandatory reporting of pediatric cardiac arrest cases, similar to the Danish model, could strengthen data quality, support national quality improvement initiatives, and facilitate long-term funding commitments.^33^, ^34^

Given the Netherlands’ small population and centralized healthcare system, findings from PROGNOSE may not fully represent other settings. Expanding pediRES-Q’s European network would allow for multicenter comparisons, fostering the development of evidence-based, multimodal neuroprognostication guidelines applicable across diverse healthcare systems. Additionally, leveraging the Dutch follow-up model could provide a blueprint for structured long-term monitoring across pediRES-Q centers. The Netherlands has one of the most structured post-ICU follow-up infrastructures, including multidisciplinary clinics and standardized neuropsychological assessments. Expanding this model within pediRES-Q could facilitate pediatric cardiac arrest survivors to receive consistent, high-quality long-term follow-up.

## Conclusion

The continued success of PROGNOSE relies on expanding participation, securing financial stability, and strengthening international collaboration. Key priorities include integrating pre-hospital OHCA non-survivors, developing structured young adult follow-up, and implementing automated data collection tools. Policy efforts supporting long-term funding and mandatory case reporting will be essential for sustainability. As the first national initiative to standardize pediatric post-ROC care and long-term follow-up in the Netherlands, PROGNOSE has the potential to drive evidence-based improvements in care and inform global best practices. Expanding data collection, ensuring lifelong follow-up, and fostering international cooperation will be crucial for maximizing its long-term impact on patient outcomes and healthcare systems.

## CRediT authorship contribution statement

**Marijn Albrecht:** Writing – original draft, Validation, Methodology, Investigation, Data curation, Conceptualization. **Maayke Hunfeld:** Writing – review & editing, Validation, Supervision, Conceptualization. **Annemieke Arkesteijn-Muit:** Investigation. **Karolijn Dulfer:** Writing – review & editing, Investigation. **Matthijs de Hoog:** Writing – review & editing, Supervision, Conceptualization. **Gabry de Jong:** Investigation. **Rogier de Jonge:** Writing – review & editing, Supervision, Methodology, Conceptualization. **Aldert Lamoré:** Investigation. **Vinay Nadkarni:** Writing – review & editing, Supervision, Conceptualization. **Corinne Buysse:** Writing – review & editing, Supervision, Methodology, Conceptualization. **Nikki Schoenmaker:** Writing – review & editing, Investigation. **Annelies van Zwol:** Writing – review & editing, Investigation. **Geanne Krabben-de Vlaam:** Writing – review & editing, Investigation. **Nicole de la Haye:** Writing – review & editing, Investigation. **Jennifer Walker:** Writing – review & editing, Investigation.

## Declaration of competing interest

The authors declare the following financial interests/personal relationships which may be considered as potential competing interests: Corinne Buysse has received funding from ZOLL Medical and the foundation “Stichting Vermeer14” for the setup of the PROGNOSE study. Both had no role in the study design, data collection, analysis, or interpretation, and will not have any influence on its outcomes or conclusions.

Vinay Nadkarni MD receives unrestricted grant funding to his institution from the National Institutes of Health, US Department of Defense, ZOLL Medical, Laerdal Foundation, RQI Partners, Philips Medical, and Nihon-Kohden. All are unrelated to this study. He serves as Editorial Board Member for Resuscitation and Pediatric Critical Care Medicine and was not involved in the editorial review or the decision to publish this article. Lastly, he serves on the Executive Committee for the Society of Critical Care Medicine (SCCM). The views expressed as an author in this manuscript are his, and are not intended to represent the views of the SCCM.
