# Supplementary material for: A Dutch nationwide pediatric cardiac arrest registry with long-term follow-up – towards an international prognostication guideline
Source: Resusc Plus. 2025 May 9;24:100976. doi: 10.1016/j.resplu.2025.100976 (PMC12148594; doi:10.1016/j.resplu.2025.100976)
Supplement: Supplementary Data 1 [file mmc1.docx]

**Supplemental files**

**Content**

[Supplemental Table 1: Post-ROC treatment protocol 2](#_Toc194764465)

[Supplemental table 2. Post-ROC prognostication protocol 3](#_Toc194764466)

[Supplemental Table 3: Overview of the outcomes assessed through interviews and patient- and parent-reported questionnaires 5](#_Toc194764467)

[Supplemental Table 4: Overview of the neurocognitive testbattery 12](#_Toc194764468)

[References 13](#_Toc194764469)

# Supplemental Table 1: Post-ROC treatment protocol

**1. Airway and Breathing**

- **Indications for Intubation**: Early intubation if no immediate return to normal cerebral function.
- **Ventilation Strategy**: Lung-protective ventilation (TV 4–6 mL/kg, PEEP 4–8 cm H₂O).
- **Oxygenation Targets**: SpO₂ 94–98%, PaO₂ 10–13 kPa (75–98 mmHg).
- **CO₂ Targets**: Normocapnia (PaCO₂ 4.5–6.0 kPa or 35–45 mmHg); avoid hypocapnia.
- **Additional**: Insert nasogastric tube, perform chest X-ray (tube/line positioning, edema, pneumothorax), and initiate sedation per protocol.

**2. Circulation and Hemodynamics**

- **Monitoring**: Arterial and central venous lines; measure CVP.
- **Targets**: SBP > 5th percentile; MAP and urine output used to guide perfusion.
- **Shock Management**: Use echocardiography to differentiate causes (e.g., distributive, cardiogenic); initiate noradrenaline ± dobutamine if vasoplegic; provide fluids if hypovolemic.

**3. Metabolic Management**

- **Glucose**: Target <10 mmol/L; avoid hypoglycemia (<2.8 mmol/L).
- **Electrolytes**: Maintain potassium 4.0–4.5 mmol/L; monitor calcium, magnesium, sodium.
- **Renal Function**: Monitor lactate clearance; assess for rhabdomyolysis.
- **Infusion Regimen**: Use isotonic saline initially; adjust glucose concentration based on age and stability.

**4. Neurology (Monitoring Only)**

- **CT Brain**: Consider early if etiology unclear (e.g., AVM, bleeding).
- **EEG**: Continuous EEG for 48h if unresponsive; use amplitude-integrated EEG if cEEG unavailable.
- **Targeted Temperature Management (TTM)**:
  - Maintain constant temperature for first 24h post-ROSC (32–36°C).
  - Avoid hyperthermia (>37.6°C) for 72h.
  - Rewarm gradually after 24h (0.25–0.5°C/h).
  - Consider magnesium infusion to reduce shivering.

**5. Gastrointestinal**

- NPO for 24 hours post-ROSC.
- Gradual reintroduction of enteral feeding during TTM.
- Consider stress ulcer prophylaxis in adolescents.

**6. Hematology**

- Deep vein thrombosis (DVT) prophylaxis in adolescents.

**7. Family-Centered Care**

- Promote open communication.
- Allow family presence during care.
- Involve multidisciplinary teams for psychosocial support.

# Supplemental table 2. Post-ROC prognostication protocol

| Day | GCS + Brainstem | EEG | CT/MRI | Biomarkers | Comments |
| --- | --- | --- | --- | --- | --- |
| 1 | Yes (initial; GCS, pupils, corneals, motor) | Start **cEEG** ideally within 12–24h (min 24–48h) | CT brain **if etiology unclear** (e.g. trauma, AVM) | Blood samples: lactate, **NSE, S100B, MBP** | Consider aEEG if no cEEG available. Avoid early prognosis decisions. |
| 2 | Repeat neuro exam | Continue cEEG monitoring (if ongoing) | — | — | Evaluate EEG background pattern; seizures, suppression. |
| 3 | Repeat neuro exam | Discontinue cEEG if stable and complete | **MRI**: Perform DWI, ADC, T1, T2 sequences | — |  |
| 4 | Repeat neuro exam | — | Optional: Repeat MRI (if first was limited/early) | — | Use all modalities together to support prognostication. |
| 5 | Repeat neuro exam | — | — |  | Rewarm complete (if under TTM); remove sedation if possible. |
| 6 | Repeat neuro exam | — | — | — |  |

**Neurological Examinations**

- Serial exams including brainstem reflexes, motor responses, and consciousness level.
- Reliable assessments only after 72 hours post-ROC, accounting for sedatives and metabolic disturbances.

**Electroencephalography (EEG)**

- **Timing**: Perform continuous EEG (cEEG) starting as early as possible, ideally within 12–24 hours post-ROSC.
- **Duration**: Minimum of 24–48 hours.
- **Classification**:
  - **Favorable**: Continuous background with amplitude ≥20 µV.
  - **Unfavorable**: Burst-suppression, isoelectric background, or low-voltage continuous (<20 µV).
- **Quantitative EEG (qEEG)**:
  - At 24h post-ROSC, qEEG features such as minimal amplitude and EEG Silence Ratio (ESR) are the most predictive of 12-month outcome.
  - A machine learning classifier trained on qEEG achieved 100% PPV for mortality prediction when classifying patients with discontinuous or low-amplitude patterns.
- **Interpretation**:
  - **Do not** rely on EEG alone for WLST.
  - Combine visual and qEEG analysis to increase predictive accuracy.

*Reference: Hunfeld et al., Neurology 2024;103:e210043*

**Magnetic Resonance Imaging (MRI)**

- **Timing**: Perform between day 3–5 post-ROSC to balance early detection and pseudonormalization risk.
- **Sequences**: Include T1, T2, DWI, and ADC maps.
- **Interpretation**:
  - **Normal MRI** (no injury): Strong predictor of favorable 2-year outcome (NPV 100%).
  - **Extensive Injury** (≥50% cortex/white matter or ≥4 brain regions involved): Strong predictor of unfavorable outcome or death (PPV 100%).
  - **Focal Injury**: Variable prognostic value; interpret in context of clinical picture and EEG.
- **Scoring**: Use a standardized scoring system to assess cortex, white matter, and deep gray matter injury.

*Reference: Albrecht et al., Pediatr Neurol 2025;165:96–104*

**Computed Tomography (CT)**

• **Timing**: Perform early if etiology of arrest is unclear (e.g., AVM, trauma, bleeding).

• **Utility**: Less sensitive than MRI for detecting hypoxic-ischemic changes but valuable in acute phase or when MRI is contraindicated.

• **Interpretation**: Use for excluding structural causes; not reliable for prognostication alone.

**Biomarkers**

• **Recommended Markers**: Neuron-Specific Enolase (NSE), S100B, Myelin Basic Protein (MBP), and Neurofilament Light (NFL).

• **Timing**: Measure serially within the first 72 hours post-ROSC.

• **Interpretation**: Supports multimodal assessment; no single cut-off should guide WLST decisions. Adjunctive use only.

**Other Notes – Multimodal Interpretation**

• Use combined input from serial clinical exams, EEG (visual and qEEG), MRI, CT, and biomarkers.

• Avoid over-reliance on single modalities.

• Prognostication should be cautious, contextual, and ideally discussed in multidisciplinary teams.

• Do not initiate WLST based on one abnormal test alone.

# Supplemental Table 3: Overview of the outcomes assessed through interviews and patient- and parent-reported questionnaires

| **Domain** | **Interview^1^** | **Questionnaire^2^** | **Informant and Questionnaires** | **Comparisons** | **Deviant score** |
| --- | --- | --- | --- | --- | --- |
| **Outcomes in children** |  |  |  |  |  |
| ***\|PICU follow-up variables*** |  |  |  |  |  |
| School missing | X |  | Number of days missed at school after hospital discharge | None |  |
|  |  |  |  |  |  |
| Re-admission | X |  | Re-admission to hospital after hospital discharge and related to PICU admission | None |  |
| Medical/psychosocial care | X |  | Question: Received medical or psychosocial care after hospital admission? | None |  |
|  |  |  |  |  |  |
| Respiratory complaints | X |  | Patient or parent reported dyspnea, tachypnea, coughing, and/or wheezing at follow-up | None |  |
| Neurological complaints | X |  | Patient or parent reported neurological symptoms at follow-up | None |  |
| Exercise intolerance | X |  | Patient or parent reported exercise intolerance: Question: Worsened exercise intolerance compared to pre hospital admission? | Individual pre-PICU |  |
| PCPC | X |  | Parent or patient reported Pediatric Cerebral Performance Category | None |  |
| POPC | X |  | Patient or parent reported Pediatric Overall Performance Category | None |  |
| Eating | X |  | Question: Worsened eating pattern compared to pre hospital admission? | Individual pre-PICU |  |
| Sleep | X |  | Question: Worsened sleep pattern compared to pre hospital admission? | Individual pre-PICU |  |
| ***Psychosocial functioning*** |  |  |  |  |  |
| Emotion/behavior |  | X | The Strengths and Difficulties Questionnaire (SDQ) parent-reported (5-17y) and self-reported (11-17y), z-scores | Dutch general population | ≤ - 1 |
| Trauma |  | X | The Children’s Revised Impact of Event Scale (CRIES-13) parent-reported (5-17y) and self-reported (8-17y) | Dutch trauma-exposed children  and adolescents | ≥ 30 |
| ***Neurocognitive functioning*** |  |  |  |  |  |
| **Neuropsychological intake** | X |  | Question: Occurred or worsened attention problems after PICU admission compared to before? | Individual pre-PICU |  |
|  | X |  | Question: Occurred or worsened memory problems after PICU admission compared to before? | None |  |
|  | X |  | Question: Occurred or worsened executive problems after PICU admission compared to before? | None |  |
|  | X |  | Question: Occurred or worsened visual spatial problems after PICU admission compared to before? | None |  |
| Cognitive functions |  | X | PROMIS Short Form V1.0 – Cognitive Function parent-reported (5-7y) and self-reported (8-17y) | Dutch general population | ≤ 45 |
| Executive functions |  | X | The Behaviour Rating Inventory of Executive Function (BRIEF) parent-reported (5-18y) and self-reported (11-17y) | Dutch general population | ≥ 65 |
| ***Quality of Life*** |  |  |  |  |  |
| Physical functioning |  | X | Pediatric Quality of Life Inventory (PedsQL); parent-reported (5-7y) and self-reported (8-17y) | Dutch general population | ≤ - 1 |
| Emotional functioning |  | X | Pediatric Quality of Life Inventory (PedsQL); parent-reported (5-7y) and self-reported (8-17y) | Dutch general population | ≤ - 1 |
| Social functioning |  | X | Pediatric Quality of Life Inventory (PedsQL); parent-reported (5-7y) and self-reported (8-17y) | Dutch general population | ≤ - 1 |
| School functioning |  | X | Pediatric Quality of Life Inventory (PedsQL); parent-reported (5-7y) and self-reported (8-17y) | Dutch general population | ≤ - 1 |
| Sleep |  | X | PROMIS® Pediatric Short Form v1.0 – Sleep-Related Impairment 8a; parent-reported (5-7y) and self-reported (8-17y) | Dutch general population | ≥ 52.5 |
| Fatigue |  | X | PROMIS Pediatric Short Form v2.0 - Fatigue 10a; parent-reported (5-7y) and self-reported (8-17y) | Dutch general population | ≥ 39.8 |
| **Psychosocial outcomes in parents** |  |  |  |  |  |
| Trauma |  | X | PTSD checklist for DSM-5 (PCL-5) | Dutch general population | ≥ 31 |
| Anxiety |  | X | PROMIS SF v1.0 – Anxiety 8a. | Dutch general population | ≥ 55.9 |
| Depression |  | X | PROMIS SF v1.0 – Depression 8b. | Dutch general population | ≥ 55.6 |
| Parental distress |  | X | Distress Thermometer for Parents (DT-P) | Dutch general population | ≥ 4 |

^1^Interview conducted with pediatric intensivist, patient and at least one parent or with psychologist as part of neuropsychological intake.

^2^Validated questionnaires were completed either on paper or digitally prior to the outpatient appointment. Each questionnaire is explained in more detail below.

**Interview with pediatric intensivist/ history taking and interview with psychologist/neuropsychological intake**

The interviews with patient and at least one parent/caregiver were conducted with a paediatric intensivist and a paediatric psychologist as part of the neuropsychological intake. All questions were asked in the presence of both patient and parent/caregiver. Depending on the child’s age (>8 years old) or the child’s developmental stage, questions were child and/or parent-reported.

In the Netherlands every Pediatric Intensive Care Unit is obligated to provide follow-up care to a subgroup of patients (mainly previously healthy children and PICU admission unplanned). The follow-up care after PICU admission has been described in the national guideline on follow-up of PICU patients (national guideline 2017, see below). Each tertiary/university hospital has implemented their own (multidisciplinairy) out-patient follow-up program based upon this guideline, however some differences between hospitals do exist. All PICUs within the Netherlands collaborated to provide an uniform and structured follow-up program as presented in Supplemental Tables 3 and 4 (Overview neurocognitive testbattery). Since these follow-up interviews, questionnaires and neurocognitive tests are described within the national guideline, it is considered as standard of care.
*Kindergeneeskunde NVvK. Richtlijn follow-up van kinderen na opname op een intensive care 2017 [Available from: https://www.nvk.nl/themas/kwaliteit/richtlijnen].*

**Patient-reported outcome measures/Questionnaires**

1. **Physical functioning**

***Sleep***

Self-reported and parent-reported sleep-related impairments were assessed with an 8 item PROMIS short form, with a recall period of 7 days on a 5-point Likert scale from never to always.

***Fatigue***

Self-reported and parent-reported fatigue was assessed with a 10 items PROMIS short form, with a recall period of 7 days on a 5-point Likert scale form never to always.

*Haverman L, Grootenhuis MA, Raat H, van Rossum MAJ, van Dulmen-den Broeder E, Hoppenbrouwers K, Correia H, Cella D, Roorda LD, Terwee CB. Dutch-Flemish translation of nine pediatric item banks from the Patient Reported Outcomes Measurement Information System (PROMIS). Qual Life Res 2016;25:761-765. (www.healthmeasures.net)*

The minimally important clinical difference (MICD) scores for PROMIS Pediatric Measures is 2 – 5 points.

*Thissen D, Liu Y, Magnus B, Quinn H, Gipson DS, Dampier C, Huang IC, Hinds PS, Selewski DT, Reeve BB, Gross HE, DeWalt DA. Estimating minimally important difference (MID) in PROMIS pediatric measures using the scale-judgment method. Qual Life Res. 2016 Jan;25(1):13-23. doi: 10.1007/s11136-015-1058-8. Epub 2015 Jun 29. PMID: 26118768; PMCID: PMC4695321.*

1. **Quality of life**

The Dutch versions of the Pediatric Quality of Life Inventory (PedsQL); parent-reported (5-7 years) and self-reported (8-17 years) were used. The PedsQL assesses perceptions of children and reflects their universal concerns on four subscales: physical (8 items), emotional (5 items), social (5 items) and

school functioning (5 items) on a 5-point Likert scale to what extent the child had difficulties with that problem. Answering options are never (0), almost never (1), sometimes (2), often (3) and almost always (4). Each answer is reversed scored and rescaled to 0-100 scale (0 = 100, 1 = 75, 2 = 50,

3 = 25 and 4 = 0). A score of 100 represents the best quality of life possible, a score of 0 the worst. The norm data consisted of 2 groups: 1) up to 12 years old, n=475 48.6% female, mean age (SD) 10.6 (1.5) years, and 2) 13 to 17 years old, n=491, 48.7% female, age mean (SD) 15.5 (1.4) years. The QoL raw scores were converted into z-scores with use of the mean and SD of the appropriate age group. The minimal important clinical differences: the PedsQL self-report 4.4 points, the PedsQL parent-report 4.5 points, and the cut-off for at risk is 1 SD below the norm population mean.

*van Muilekom MM, Luijten MAJ, van Oers HA, Conijn T, Maurice-Stam H, van Goudoever JB, Grootenhuis MA, Haverman L; KLIK collaborator group. Paediatric patients report lower health-related quality of life in daily clinical practice compared to new normative PedsQLTM data. Acta Paediatr. 2021 Jul;110(7):2267-2279. doi: 10.1111/apa.15872.*

*Varni JW, Burwinkle TM, Seid M. The PedsQL as a pediatric patient-reported outcome: reliability and validity of the PedsQL Measurement Model in 25,000 children. Expert Rev Pharmacoecon Outcomes Res. 2005 Dec;5(6):705-19. doi: 10.1586/14737167.5.6.705. PMID: 19807613.*

1. **Emotional and behavioral functioning**

***Emotional and behavioural problems***

The Dutch versions of the Strengths and Difficulties Questionnaire – Parent Form (parents regarding their child 4-17 years) and Child Form (self-report by children ≥ 11 years) were used. The SDQ consists of 25 items, scored on a Likert scale from 0 (not true) to 2 (certainly true) divided in 5 scales: Emotional symptoms, Conduct problems, Hyperactivity-Inattention, Peer problems and Prosocial behaviour. Higher scores on all scales, except the Prosocial behaviour scale, reflect difficulties. A higher score on the Prosocial behaviour scale reflects strength.

The parent-reported SDQ raw scores were converted into z-scores with use of the mean and SD of the appropriate group (6 to 11 years boys, 6 to 11 years girls, 12 to 18 years boys, 12 to 18 years girls) [Maurice-Stam_2018]. The self-reported SDQ scores were compared with that of Dutch adolescents norm scores [Vugteveen et al. 2021]

*Maurice-Stam H, Haverman L, Splinter A, van Oers HA, Schepers SA, Grootenhuis MA. Dutch norms for the Strengths and Difficulties Questionnaire (SDQ) - parent form for children aged 2-18 years. Health Qual Life Outcomes. 2018;16(1):123.*

*Vugteveen J, de Bildt A, Theunissen M, Reijneveld M, Timmerman M. Validity Aspects of the Strengths and Difficulties Questionnaire (SDQ) Adolescent Self-Report and Parent-Report Versions Among Dutch Adolescents. Assessment. 2021 Mar;28(2):601-616.*

***Posttraumatic stress disorder (PTSD)***

The Dutch versions of The Children’s Revised Impact of Event Scale (CRIES-13) parent version (parents regarding child 5-17 years) and child version (children ≥ 8 years regarding themselves) were used to assess PTSD in children. The CRIES-13 consists of 13 items, scored on a 4 point Likert scale from ‘not at all’ to ‘often’ with a recall period of 7 days. It consists of 3 subscales, intrusion, avoidance, and arousal, and 1 total score. A total core of 30 or higher indicates risk for PTSD [Verlinden et al. 2014]. The risk for PTSD was compared with the prevalence of PTSD a trauma-exposed children and adolescents [Alisic et al_ 2014].

*Verlinden et al. (2014). A Parental Tool to Screen for Posttraumatic Stress in Children: First Psychometric Results. Journal of Traumatic Stress, 27, 1-4. Verlinden et al. Characteristics of the Children’s Revised Impact of Event Scale in a Clinically Referred Dutch Sample, 2014*

*Alisic E, Zalta AK, van Wesel F, Larsen SE, Hafstad GS, Hassanpour K, Smid GE. Rates of post-traumatic stress disorder in trauma-exposed children and adolescents: meta-analysis. Br J Psychiatry. 2014;204:335-40. doi: 10.1192/bjp.bp.113.131227. PMID: 24785767.*

1. **Cognitive functioning**

***Executive function***

Executive functioning was assessed with the Behavior Rating Inventory of Executive Function in children aged 2 years 6 months - 5 years 11 months with BRIEF-P, and in children 6 years – 17 years 11 months with BRIEF, filled out by the parents/caregivers of the child. Overlapping scales and indices of both questionnaires (Inhibition, Flexibility, Emotional Control, Working Memory, Planning and Organization, Meta-cognition) and a Total Score were analyzed (T-scores, with mean 50 and SD 10). A T-score of 65 or higher is interpreted as an abnormally elevated score.

*Van der Heijden KB, Suurland J, De Sonneville LM, et al. BRIEF-P Vragenlijst voor executieve functies voor 2- tot 5-jarigen: Handleiding. Amsterdam: Hogrefe 2013.*

*Huizinga M, Smidts D. BRIEF Vragenlijst executieve functies voor 5- tot 18-jarigen: Handleiding. Amsterdam: Hogrefe, 2012.*

***Cognitive function***

Parent- or self-reported cognitive function was assessed with the PROMIS Pediatric Short Form v1.0 – Cognitive Function 7a (self-reported 8 – 18 years) or PROMIS Parent Proxy Short Form v1.0 – Cognitive Function 7a (parent-reported 5-7 years). This questionnaires consists of 7 items, with a recall period of 4 weeks, with a 5-points answer scale from never to always. The minimally important clinical difference (MICD) scores for PROMIS Pediatric Measures is 2 – 5 points.

*Thissen D, Liu Y, Magnus B, Quinn H, Gipson DS, Dampier C, Huang IC, Hinds PS, Selewski DT, Reeve BB, Gross HE, DeWalt DA. Estimating minimally important difference (MID) in PROMIS pediatric measures using the scale-judgment method. Qual Life Res. 2016 Jan;25(1):13-23. doi: 10.1007/s11136-015-1058-8. Epub 2015 Jun 29. PMID: 26118768; PMCID: PMC4695321.*

*Lai, J-S., Zelko, F., Krull, K., Cella, D., Nowinski, C., Manley, P., Goldman, S. (2014). Parent-reported cognition of children with cancer and its potential clinical usefulness. Quality of Life Research, 23, 1049-1058***Outcomes in parents**

***Posttraumatic stress disorder (PTSD)***

PTSD checklist for DSM-5 (PCL-5) screens for symptoms of posttraumatic stress disorder (PTSD), with a 1-month recall period, based on the Diagnostic and Statistical manual of Mental disorders (DSM-IV) criteria [Boeschoten et al.]. The PCL-5 includes 20 items, scored on a Likert scale ranging from 0 to 4, reflecting the DSM-5 diagnostic criteria of PTSD. A higher scores represents more pronounced PTSD symptoms and a cut-off score of 31 was used to screen for PTSD (specifity of .95 sensitivity of .85). Since Dutch norm scores are lacking, the prevalence of 7% PTSD in the general Dutch adult population was used to compare with: [*https://www.vzinfo.nl/posttraumatische-stressstoornis*](https://www.vzinfo.nl/posttraumatische-stressstoornis)

*Boeschoten, M.A., Bakker, A., Jongedijk, R.A. & Olff, M. (2014). PTSD Checklist for DSM-5– Nederlandstalige versie. Uitgave: Stichting Centrum ’45, Arq Psychotrauma Expert Groep, Diemen.*

***Anxiety and Depression***

The PROMIS SF v1.0 – Anxiety 8a and the PROMIS SF v1.0 – Depression 8b short-forms were used to assess symptoms of respectively anxiety and depression in parents. Both measures comprise of 8 items, scored on a Likert scale ranging from ‘never’ to ‘almost’ using a 7-day recall period. Total scores are calculated and transformed into a T-score with mean 50 and SD10. Higher scores mean more anxiety or depression symptoms. Dutch PROMIS norm scores were used to compare [Elsman et al.]. The minimally important clinical difference (MICD) scores for PROMIS Measures is 2 – 6 points.

*Terwee CB, Roorda LD, de Vet HCW, Dekker J, Westhovens R, van Leeuwen J, Cella D, Correia H, Arnold B, Perez B, Boers M. Dutch-Flemish translation of 17 item banks from the Patient Reported Outcomes Measurement Information System (PROMIS). Quality of Life Research 2014;23:1733-1741.*

*Elsman EBM, Flens G, de Beurs E, Roorda LD, Terwee CB. Towards standardization of measuring anxiety and depression: Differential item functioning for language and Dutch reference values of PROMIS item banks. Submitted for publication.*

*Terwee CB, Peipert JD, Chapman R, Lai JS, Terluin B, Cella D, Griffith P, Mokkink LB. Minimal important change (MIC): a conceptual clarification and systematic review of MIC estimates of PROMIS measures. Qual Life Res. 2021 Oct;30(10):2729-2754. doi: 10.1007/s11136-021-02925-y. Epub 2021 Jul 10. PMID: 34247326; PMCID: PMC8481206.*

***Parental distress***

Parental distress in parents was measured with the “Distress Thermometer for Parents” (DT-P) (Haverman et al. 2013). The DT-P consists of a thermometer score, regarding overall distress (0 no distress to 10 extreme distress), with a cut-off of 4 indicating clinically relevant. The norm group consisted of 1421 parents (60,7 % mothers) of children in the Dutch general population (aged 8.1 (5.6)). Results of parental distress were compared to Dutch reference parents of healthy children (van Oers et al. 2017)

*Haverman L, van Oers HA, Limperg PF, Houtzager BA, Huisman J, Darlington AS, Maurice-Stam H, Grootenhuis MA. Development and validation of the distress thermometer for parents of a chronically ill child. J Pediatr. 2013 Oct;163(4):1140-6.e2.*

*van Oers HA, Schepers SA, Grootenhuis MA, Haverman L. Dutch normative data and psychometric properties for the Distress Thermometer for Parents. Qual Life Res. 2017 Jan;26(1):177-182. doi: 10.1007/s11136-016-1405-4. Epub 2016 Sep 2. PMID: 27589979; PMCID: PMC5243897.*

# Supplemental Table 4: Overview of the neurocognitive testbattery

|  | **Test** | **Age (yrs.)** | **Test mean and**  **standard deviation (SD)** | **Notes** | **Deviant score** |
| --- | --- | --- | --- | --- | --- |
| General intelligence | Wechsler Preschool and Primary Scale of Intelligence (WPPSI-III) | 2.6-6 | Mean 100, SD 15 [2] | Intelligence quotient standard scores. Higher scores represent better functioning. Total IQ score is based on WPPSI-III TIQ, WISC-V TIQ, or WAIS-IV TIQ. The Verbal Comprehension index is based on: WPPSI-III VIQ, WISC-V VCI, WAIS-IV VCI.  Deviant scores are interpreted as ≤ 85 | ≤ 85 |
|  | Wechsler Intelligence Scale for Children (WISC-V) | 7-15 | Mean 100, SD 15 [3] |  |  |
|  | Wechsler Adult Intelligence Scale (WAIS-IV) | 16-18 | Mean 100, SD 15 [4] |  |  |
| Verbal memory:  immediate recall | Rey auditory verbal learning test (Rey-AVLT) | ≥6 | Mean 0, SD1[8, 9] | Z-score compared with age appropriate scores: Higher scores represent better functioning | ≤ - 1 |
| Verbal memory:  delayed recall | Rey auditory verbal learning test (Rey-AVLT) | ≥6 | Mean 0, SD1[8, 9] | Z-score compared with age appropriate scores: Higher scores represent better functioning |  |
| Selective attention | Stroop Color Word Test (Stroop) | ≥11 | Mean 0, SD1 [5] | Z-score; Higher scores represent better functioning | ≤ - 1 |
| Sustained attention | Bourdon Vos cancellation test | ≥6 | Mean 0, SD1 [6] | Z-score compared with age appropriate scores; Higher scores represent better functioning | ≤ - 1 |
| Visual-Motor Integration | Beery Developmental Test of Visual Motor Integration (Beery-VMI) | ≥2 | Mean 0, SD1 [7] | Z- score: Higher scores represent better functioning | ≤ - 1 |
| Visual memory:  3 minutes recall | Rey-Osterrieth Complex Figure test (Rey CFT) | ≥6 | Mean 0, SD1 [10] | Z-score: Higher scores represent better functioning | ≤ - 1 |
| Visual memory:  30 minutes recall | Rey-Osterrieth Complex Figure test (Rey CFT) | ≥6 | Mean 0, SD1 [10] | Z-score: Higher scores represent better functioning | ≤ - 1 |
| Executive functions: flexibility | Trail Making Test part B (TMT-B) | ≥8 | Mean 0, SD1 [11] | Z-score compared with age appropriate scores: Higher scores represent better functioning | ≤ - 1 |
| Executive functions: Strategy formation | Behavioural Assessment of the Dysexecutive Syndrome in Children  (BADS-C) key search | ≥8 | Mean 0, SD1 [1] | Z-score: higher scores represents better functioning. | ≤ - 1 |
| Executive functions: Planning | Behavioural Assessment of the Dysexecutive Syndrome in children  (BADS-C) six parts test | ≥8 | Mean 0, SD1 [1] | Z-score: higher scores represents better functioning. | ≤ - 1 |

# References

1. Emslie H, F. C. Wilson, V. Burden, I. Nimmo-Smith & B.A. Wilson Nederlandse vertaling: A.C. Tjeenk-Kalff & L. Krabbendam BADS-C l Behavioural Assessment of the Dysexecutive Syndrome for Children Ecologisch valide testbatterij voor executief functioneren bij kinderen van 8 tot 16 jaar.

2. Hendriksen J, Hurks P (2009) WPPSI-III NL. Wechsler Preschool and Primary Scale of Intelligence - Third Edition. Nederlandstalige bewerking. Afname- en scoringshandleiding. [Dutch version of the WPPSI-III]. Pearson, Amsterdam

3. Marc P.H. Hendriks & Selma Ruiter (2019) WISC-III NL Wechsler Intelligence Scale for Children-V. Vijfde Editie NL. Handleiding en Verantwoording [Dutch version of the WISC-V]. Pearson Clinical Assessment.

4. Wechsler D (2012) Wechsler adult intelligence scale – fourth edition. Nederlandstalige bewerking. [Dutch version of the WAIS-IV]. Pearson Assessment and information B.V., Amsterdam

5. Schmand B, Houx P, de Koning I (2005) Normen voor Stroop Kleurwoord Tests, Trail Making Test en Story Recall van de Rivermead Behavioral Memory Test [Dutch reference data for the Stroop, TMT]. Sectie Neuropsychologie, Nederlands Centrum voor Psychologen, Amsterdam,

6. Vos P. (1998) Bourdon Vos Test, Nederlandse editie. Pearson, Amsterdam,

7. Beery KE, Beery NA (2004) The Beery-Buktenica developmental test of visual-motor integration (5th ed.). NCS Pearson Inc, Minneapolis, MN

8. Schmand B, Houx P, de Koning I (2012) Normen 15-Woordentest [Dutch reference data for the RAVLT]. sectie Neuropsychologie, Nederlands Instituut van Psychologen, Amsterdam

9. Kok TB, Kingma A, (2009) Herkenningsgeheugen bij kinderen [Dutch reference data for the RAVLT]. Tijdschrift voor Neuropsychologie 4: 42-49

10. Rey A (1964) L'examen clinique en psychologie. Presses Universitaires de France, Paris

11. Strauss E, Sherman EMS, Spreen O (2006) A compendium of neuropsychological tests : administration, norms, and commentary. Oxford University Press, Oxford; New York

12. Smidts D, Huizinga M (2009) BRIEF : executieve functies gedragsvragenlijst [Dutch version of the BRIEF]. Hogrefe Uitgevers, Amsterdam.
