# Supplementary material for: A Dutch nationwide pediatric cardiac arrest registry with long-term follow-up – towards an international prognostication guideline
Source: Resusc Plus. 2025 May 9;24:100976. doi: 10.1016/j.resplu.2025.100976 (PMC12148594; doi:10.1016/j.resplu.2025.100976)
Supplement: Supplementary Data 2 [file mmc2.pdf]

Pediatric Resuscitation Neuroprognostication and Outcomes Registry (PROGNOSE) - version 325.31

Pediatric Resuscitation Neuroprognostication and Outcomes Registry

Printed on 05-04-2025 16:14:18 by Marijn Albrecht

1. Lifeliner Pre-Hospital Data - Lifeliner Pre-Hospital Data

Number	Question	Answers
1.1	Date and Time of Arrest<br>Earliest time that the need for chest compressions was first recognized by direct observation. ***If time is unknown, enter 00:00***.	___ ___ ___ *(dd-mm-yyyy)*<br>___ : ___ *(hh:mm)*
1.2	Attending Lifeliner	○ Lifeliner 1<br>○ Lifeliner 2<br>○ Lifeliner 3<br>○ Lifeliner 4
1.3	Type of deployment<br>Specify the type of deployment.	○ Primary deployment<br>○ Secondary deployment<br>○ Rendez-vous<br>○ Deployment to secondary care hospital
1.4	Location of Event<br>Select where the resuscitation occurred: either in a private home/space or in a public facility (work, street, public building, sport event).	○ Home/Private space<br>○ Public space<br>○ Other
1.5	Postal code of deployment<br>Enter the four digits and two letters of the Dutch postal code of the deployment site ("1234AB"). For sites that are NOT allowed to disclose this per regulatory restrictions and/or if postal code is unknown, enter 0000.	___
1.6	Date of birth<br>Enter the patient's date of birth. DD-MM-YYYY. For sites that are NOT allowed to disclose ANY dates (date of arrest and/or date of birth) per regulatory restrictions, you can leave this field blank, but must MANUALLY enter AGE.	___ ___ ___ *(dd-mm-yyyy)*
1.7	Age at Arrest<br>*Warning shown if field's value is larger than 17.99: 'The entered age is not in accordance with the definition for inclusion, which defines a maximum age of 17.99.'*<br>Enter age manually as follows: Enter as YEAR(S) and/or fraction of year rounded to the nearest hundredth, i.e., patient is 6 months and 12 days old = "0.54"; a patient that is 9 days old = 0.025 -> enter as "0.03". Enter in format y.xx where y is 1 or more digits and xx is always 2 digits.	___ years
1.8	Gender<br>The patient's documented sex.	○ Female<br>○ Male
1.9	Characterize outcome of event<br>Applicable to pediatric out-of-hospital cardiac arrests (≥ 1 minute of CPR either by bystander lay-person or EMS and age < 18 years). Either CPR was discontinued at scene and patient was pronounced deceased at scene or patient was transported to a hospital with ROC or ongoing CPR. **"ROSC"**: Patient attained ROSC (Return of Spontaneous Circulation) for more than 20 minutes and no longer required chest compressions. **"ROC with ECMO"**: Patient was transferred to ECMO during the resuscitation event and no longer required chest compressions.	○ Patient pronounced deceased at scene<br>○ (Accompanied) Transport to (academic) hospital with ROC<br>○ (Accompanied) Transport to (academic) hospital with ongoing CPR
1.10	Immediate cause(s) of CPR event: (select all that apply)<br>Select primary cause resulting in cardiac arrest.	☐ ALTE/SIDS<br>☐ Airway obstruction or displacement<br>☐ Arrhythmia<br>☐ Drowning<br>☐ Electrolyte abnormality<br>☐ Elevated ICP<br>☐ Hypotension/shock<br>☐ Ingestion/Toxin<br>☐ Other respiratory failure (e.g. hypoxic)<br>☐ Seizures<br>☐ Trauma<br>☐ Other<br>☐ Unknown / Not documented
1.10.1	***If 'Immediate cause(s) of CPR event: (select all that apply)' is equal to 'Other' answer this question:***<br>Immediate cause - Other Specify<br>Specify other immediate cause(s) of the CPR event.	___
1.11	Relationship between Medical History and Cause of Arrest<br>Enter the most appropriate option. • **Medical history and cause of arrest unrelated** – Patient did not have any medical history or medical history and event cause are clearly unrelated (for example: trauma is the arrest cause and medical history of renal insufficiency). • **Medical history was the cause of arrest** – Patient diagnosed with the disease that is the cause of arrest (for example: arrest cause is long-QT syndrome and patient had been diagnosed prior to arrest) . • **Medical history could have been cause of arrest** – Patient who is diagnosed with a disease and it could have been the arrest cause (for example: patient is diagnosed with a primary arrythmogenic disorder prior to arrest and arrest rhythm was shockable but the rhythm strip is not available) • **Unknown if related** – Patient has a medical history but the arrest cause is unknown or it is unclear whether medical history and arrest cause are related.	○ Medical history (also if no medical history) and cause of arrest are unrelated<br>○ Medical history was the cause of arrest<br>○ Medical history could have been cause of arrest<br>○ Unknown if related
1.12	Witnessed Arrest<br>**Yes** - If the onset of the cardiopulmonary arrest was directly observed by someone (family, lay bystander, employee, or health care professional).	○ Yes<br>○ No<br>○ Unknown/Not documented

1.13	Bystander CPR<br>**Yes** - If a lay person performed CPR prior to EMS arrival or transport to the hospital. Select "Unknown/Not Documented" if this is not explicitly observed, stated or documented.	○ Yes<br>○ No<br>○ Unknown / Not documented
1.14	Time Chest Compressions Started<br>Enter the time that chest compressions began either by CPR monitoring if the pads were on for the first chest compression, or by direct observation. (HH:MM, military time). ***If time is unknown, enter 00:00***	___ : ___ *(hh:mm)*
1.15	Was an AED attached to the patient?<br>Yes - If an Automated External Defibrillator (AED) was used either by a civilian, text message system alerted lay person or police/fire fighter in the field. 'Use' in this question means attached to the patient and is independent whether a shock was advised or not.	○ Yes<br>○ No<br>○ Unknown/Not Documented
1.15.1	***If 'Was an AED attached to the patient?' is equal to 'Yes' answer this question:***<br>Who provided the AED?<br>Select who provided the AED. If unsure or unknown select this option AS WELL, other people can still be selected for example if it is certain that EMS provided CPR but uncertain if a parent provided CPR prior to EMS arriving. 'AED use' in this question means attached to the patient and is independent whether a shock was advised or not.	☐ Parent/Caregiver<br>☐ Bystander<br>☐ Message alerted lay-rescuer<br>☐ Police<br>☐ Firefighter<br>☐ Unsure/Unknown
1.16	Time EMS arrived at scene<br>Enter the time that the first EMS (ambulance or MMT/HEMS) arrived. (HH:MM, military time). If no bystander BLS was given, time of EMS arrival can occur before time chest compressions started. ***If time is unknown, enter 00:00***	___ : ___ *(hh:mm)*
1.17	Who performed CPR?<br>Select all people that performed CPR. If unsure or unknown select this option AS WELL, other people can still be selected for example if it is certain that EMS provided CPR but uncertain if a parent provided CPR prior to EMS arriving.	☐ Parent/Caregiver<br>☐ Bystander<br>☐ Message alerted lay-rescuer<br>☐ Police<br>☐ Firefigher<br>☐ EMS<br>☐ Unsure/Unknown
1.18	Initial rhythm when compressions started<br>Select the rhythm when compressions first started. If the patient is un-monitored, select the first rhythm when monitor became available. • Select "Unknown" if no rhythm was ever documented during the CPR event.	○ Shockable rhythm<br>○ Non-shockable rhythm<br>○ Unknown
1.19	Number of Shocks<br>Enter the number of shocks administered to the patient during the resuscitation (PREHOSPITAL) either by AED or defibrillator. If the number of shocks was unknown or not documented, enter 0.00.	______
1.20	Non-Drug Interventions by MMT/HEMS<br>Select all that apply. If "Other" is selected, describe the intervention below.	☐ None<br>☐ Endotracheal intubation<br>☐ Echocardiogram (intra-arrest)<br>☐ Cardiopulmonary bypass/ extracorporeal CPR (eCPR)<br>☐ Chest tube(s) inserted<br>☐ Needle thoracostomy<br>☐ Pacemaker, transcutaneious<br>☐ Pacemaker, transvenous or epicardial<br>☐ Pericardiocentesis<br>☐ Other Non-Drug Interventions (e.g. intraosseous infusion)
1.20.1	***If 'Non-Drug Interventions by MMT/HEMS' is equal to 'Other Non-Drug Interventions (e.g. intraosseous infusion)' answer this question:***<br>Non-Drug Interventions by MMT/HEMS - Other<br>Specify other interventions performed.	______
1.9.1	***If 'Characterize outcome of event' is not equal to 'Patient pronounced deceased at scene' answer this question:***<br>Time EMS leaving the scene<br>Enter the time that EMS treated the patient at the scene (i.e. time of leaving the scene with the patient/treatment interval) (HH:MM, military time). ***If time is unknown, enter 00:00***	___ : ___ *(hh:mm)*
1.9.2	***If 'Characterize outcome of event' is not equal to 'Patient pronounced deceased at scene' answer this question:***<br>Time EMS arrived at hospital<br>Enter the time that EMS arrived at the hospital (HH:MM, military time). ***If time is unknown, enter 00:00***	___ : ___ *(hh:mm)*
1.21	Total CPR duration: Total minutes of CPR for this event (min). If unsure, provide an educated estimate rounded UP to the nearest 5-minute interval.<br>Indicate total time of CCs from the first CC to the beginning of sustained ROSC (>20 minutes) or death. Calculate total duration of CPR given during this resuscitation event. Should include both out of hospital and in hospital (if applicable) CPR time. Use direct observation, video monitoring, or CPR data to calculate the total duration of CPR. • If ROSC was attained for less than 20 minutes and chest compressions resumed, enter all epochs of chest compressions required during this event. • The event does not end until ROSC lasts >20 minutes. • If unsure, provide an educated estimate rounded UP to the nearest 5-minute interval.	______ minute(s)
1.9.3	***If 'Characterize outcome of event' is equal to 'Patient pronounced deceased at scene' answer this question:***<br>Reason for discontinuation of CPR at scene<br>If CPR was discontinued at scene, how could the reason best be characterized.	○ Clear signs of death<br>○ Child with palliative treatment code<br>○ Cause of arrest (e.g. extent of injury or submersion time)<br>○ Duration of CPR<br>○ Other
1.9.3.1	***If 'Reason for discontinuation of CPR at scene' is equal to 'Other' answer this question:***<br>Reason for discontinuation of CPR at scene - Other<br>Specify other reason for discontinuation of CPR at scene.	______

2. Demographic and Patient Characteristics - Demographic and Patient Characteristics

Number	Question	Answers
	**IMPORTANT: In case of event survival (or the expectation of survival to hospital discharge) informed consent needs to be asked within 14 days after the screening date. This date is the date of the arrest event. In case a child does not survive the arrest, no informed consent is needed for data entry and question 2.1 can be skipped.**	
2.1	Date of Arrest<br>Enter the date of cardiac arrest. If the patient had multiple arrests, use the date of the arrest for which you have CPR data. If there are more than one, use the first event. DD-MM-YYYY. • For sites that are NOT allowed to disclose ANY dates (date of arrest and/or date of birth) per regulatory restrictions, please enter modified date of arrest as the first day of the month of arrest, i.e., "01 Oct 2016" for any arrest in the month of October 2016.	*(dd-mm-yyyy)*
2.2	Date and Time of Hospital Admission<br>As documented in the patient's chart or based on best estimate. Admission for the current university hospital visit. DD-MM-YYYY, HH:MM, military time. • If this is an in-hospital cardiac arrest, date and time this patient was admitted to the inpatient service. • If this is an out of hospital cardiac arrest, and the patient arrived to the hospital with CPR in progress or ROSC, date and time patient accepted to the emergency department.	*(dd-mm-yyyy)*<br>: *(hh:mm)*
2.3	Date and Time of ICU Admission<br>As documented in the patient's chart for the current hospital visit or based on best estimate. DD-MM-YYYY, HH:MM, military time. If this is an in-hospital cardiac arrest, date and time this patient was most recently admitted to ICU. Can be before or after the event.	*(dd-mm-yyyy)*<br>: *(hh:mm)*
2.4	Date of birth<br>Enter the patient's date of birth. DD-MM-YYYY. For sites that are NOT allowed to disclose ANY dates (date of arrest and/or date of birth) per regulatory restrictions, you can leave this field blank, but must MANUALLY enter AGE.	*(dd-mm-yyyy)*
2.5	Age at Arrest (Auto-calculated from DOB, above)<br>*Warning shown if field's value is larger than 17.99: 'The entered age is not in accordance with the definition for inclusion, which defines a maximum age of 17.99.'*<br>If you entered the patient's date of birth, this is automatically calculated and you can skip the manual entry of the age.	
2.6	Age at Arrest<br>*Warning shown if field's value is larger than 17.99: 'The entered age is not in accordance with the definition for inclusion, which defines a maximum age of 17.99.'*<br>Enter age manually as follows: Enter as YEAR(S) and/or fraction of year rounded to the nearest hundredth, i.e., patient is 6 months and 12 days old = "0.54"; a patient that is 9 days old = 0.025 -> enter as "0.03". Enter in format y.xx where y is 1 or more digits and xx is always 2 digits.	years
2.7	Gender<br>The patient's documented sex upon arrival to the hospital.	○ Female<br>○ Male
2.8	Postal code of parents<br>Enter the four digits and two letters of the Dutch postal code of the parents ("1234AB"). For sites that are NOT allowed to disclose the full postal code per regulatory restrictions enter the four digits only and if postal code is unknown or can not be shared, enter 0000.	
2.9	Weight<br>Document the patient's weight in kilograms (kg) at the time of the arrest, or the closest time point to the arrest. Enter 00.00 if the weight is not recorded or not available.	kg
2.10	Has this patient had a previous or subsequent arrest that is entered in the PROGNOSE database?<br>Indicate if this patient had an additional record(s) (in- or out-of-hospital) that have been, or will be, entered into Castor. Please try to enter the record number if known so patient can be linked to all record, even if not the same hospital admission. **Yes** - Select and go to next question and enter record number. **No** - Select if this is the only known entered record entered into Castor for this patient.	○ Yes, as specified below<br>○ No, this is only entered event for this patient
2.10.1	***If 'Has this patient had a previous or subsequent arrest that is entered in the PROGNOSE database?' is equal to 'Yes, as specified below' answer this question:***<br>If yes, please indicate Castor record number (can enter more than one record number, if applicable)<br>Enter Castor record number(s) assigned to the patient's other arrest event(s) (in- or out-of-hospital). Seperate multiple numbers with a semicolon. **Do not lead entry with a "-" [dash] (will export as an error).**	
2.11	Previous Arrest (select all that apply)<br>**• No** - If this is the patient's first cardiac arrest, select "None". **• Yes** - If the patient has had previous cardiac arrests, select all that apply: **• During current admission**: **Select "In-hospital, this admission"**, if the patient had a cardiac arrest in this hospital over the course of their current admission. **• Prior to this admission**: **Select "In-hospital (other hospital) immediately prior to this admission"**, if the patient had a cardiac arrest in a hospital setting prior to direct transfer to data collection institution. **Select "Out of hospital prior to this admission",** if the patient had an arrest out of the hospital setting that directly relates to their current hospital admission. **• Not prior to this current admission**: **Select "In-hospital, not prior to this admission"**, if the patient had a previous arrest in the hospital setting, but is before their current hospital admission. **Select "Out of hospital, not prior to this admission"**, if the patient had an arrest out of the hospital setting and has since recovered. This should not be related to this hospital admission.	☐ None<br>☐ In-hospital - this admission<br>☐ In-hospital (transported from other hospital) - immediately prior to this admission<br>☐ In-hospital, not associated with this admission<br>☐ Out of hospital - immediately prior to this admission<br>☐ Out of hospital, not associated with this admission<br>☐ Unknown / Not documented
2.12	Gestational age at patient's birth in weeks<br>Provide gestational age of the patient at the time of their birth in completed weeks. If the GA is uncertain, please provide your best estimate or indicate unknown by entering "99".	weeks
2.13	Patient's birth weight in gram<br>Provide the patient's birth weight in gram. If uncertain, please provide your best estimate or indicate unknown by entering "99".	gram
	**Comments**	
2.14	Comments<br>**Do not lead entry with a "-" [dash] (will export as an error).**	

3. Medical History and Cause of Arrest - Medical History and Cause of Arrest

Number	Question	Answers
	Medical History	
3.1	Pre-existing Conditions (select all that apply)<br>Select all options that were documented in the patient's medical record prior to the arrest. This If "Other" is selected, describe below. Note: This list is different from pre-existing conditions - at time of CPR event, please review carefully.	☐ None<br>☐ Asthma<br>☐ Cancer<br>☐ Cardiac Malformation (e.g. corrected or pre-operative congenital HD)<br>☐ Chronic lung disease<br>☐ Congenital Malformation (Non Cardiac)<br>☐ Developmental delay/mental retardation<br>☐ Diabetes<br>☐ Epilepsy<br>☐ Ex-prematurity<br>☐ Hepatic insufficiency<br>☐ Immunodeficiency (including HIV)<br>☐ Metabolic/Electrolyte Abnormality<br>☐ Myocardial dysfunction (e.g. cardiomyopathy)<br>☐ Neuromuscular disease<br>☐ Neurosurgical procedure (brain)<br>☐ Renal insufficiency<br>☐ Tracheal canule or mechanical ventilation<br>☐ Ventriculostomy/ventricular shunt<br>☐ Other (e.g. arrhythmia)
3.1.1	***If 'Pre-existing Conditions (select all that apply)' is equal to 'Cancer' answer this question:***<br>What type of cancer (select all that apply)<br>If the patient has or had previously diagnosed cancer, select the type that best applies.	☐ Hematologic<br>☐ Solid<br>☐ Bone Marrow Transplant<br>☐ Brain tumor<br>☐ Unknown
3.2	Pre-existing Conditions Other and/or Specifics<br>Specify either other pre-existing conditions if selected or elaborate on selected pre-existing conditions. For example: when congenital malformation is selected specify the condition; "congenital diaphragmatic hernia".	
	Cause of Arrest	
3.3	Pre-existing conditions - at time of CPR event (Select only conditions that existed within 4 hours prior to the event).<br>Select only conditions that existed within 4 hours prior to the event. For those conditions where there is a time interval indicated, only respond affirmatively if the diagnosis is made prior to the event for which you are completing the event form. There is no limit on the number of conditions that you can select, so you should select all of the conditions that apply.	☐ None<br>☐ Cardiac Malformation<br>☐ Congenital Malformation (Non Cardiac)<br>☐ Congestive heart failure<br>☐ Hypotension/Hypoperfusion<br>☐ Major Trauma<br>☐ Metabolic/Electrolyte Abnormality<br>☐ Metastatic or Hematologic Malignancy<br>☐ Pneumonia<br>☐ Renal insufficiency<br>☐ Respiratory insufficiency<br>☐ Septicemia<br>☐ Chromosomal/genetic abnormality<br>☐ Other
3.3.1	***If 'Pre-existing conditions - at time of CPR event (Select only conditions that existed within 4 hours prior to the event).' is equal to 'Metastatic or Hematologic Malignancy' answer this question:***<br>What type of cancer (select all that apply)<br>If the patient has previously diagnosed cancer, select the type that best applies.	☐ Hematologic<br>☐ Solid<br>☐ Bone Marrow Transplant<br>☐ Brain tumor<br>☐ Unknown
3.4	Pre-existing condition Other and/or Specifics - at time of CPR event<br>Specify either other pre-existing conditions if selected or elaborate on selected pre-existing conditions at time of CPR event. For example: when congenital malformation is selected specify the condition; "congenital diaphragmatic hernia".	
3.5	Congenital Heart Disease (pre- or post-operative)?<br>**Yes** - If documented in the patient's record prior to the event. Includes the following: • Aortic Valve Stenosis (AVS) • Patent Ductus Arteriosus (PDA) • Atrial Septal Defect (ASD) • Pulmonary Atresia • Coarctation of the Aorta (CoA) • Pulmonary Valve Stenosis • Complete Atrioventricular Canal defect (CAVC) • Single Ventricle Defects • D-transposition of the Great Arteries • Tetralogy of Fallot • Ebstein's Anomaly • Total Anomalous Pulmonary Venous Return • Hypoplastic Left Heart Syndrome • Tricuspid Atresia • Interrupted aortic arch • Truncus Arteriosus • Ventricular Septal Defect (VSD).	○ Yes<br>○ No
3.5.1	***If 'Congenital Heart Disease (pre- or post-operative)?' is equal to 'Yes' answer this question:***<br>Single ventricle?<br>**Yes** - if the patient has a previously documented congenital heart defect affecting one ventricle. These are characterized by disorders affecting one lower chamber of the heart. The chamber may be smaller, underdeveloped, or missing a valve.	○ Yes<br>○ No

3.5.2	***If 'Congenital Heart Disease (pre- or post-operative)?' is equal to 'Yes' answer this question:***<br>For those with congenital heart disease: Specify anatomy at time of CPR event. Select all as documented in patient's medical record.<br>Select all that apply.	☐ Bi-directional Glenn (Hemi-Fontan)<br>☐ Fontan<br>☐ Hybrid<br>☐ Norwood with modified BT Shunt<br>☐ Norwood with Sano Modification<br>☐ Pre-operative<br>☐ Tetralogy of Fallot<br>☐ Other (if not characterized above, e.g. corrected VSD))
3.5.2.1	***If 'For those with congenital heart disease: Specify anatomy at time of CPR event. Select all as documented in patient's medical record.' is equal to 'Other (if not characterized above, e.g. corrected VSD))' answer this question:***<br>Other: Please describe other cardiac anatomy if not specified above.	
3.6	Illness Category<br>Enter the most appropriate illness category at the time of the event onset. • **Medical-Cardiac** – Patient with a primary diagnosis of medical illness that is cardiovascular at the time of the event. • **Medical-Noncardiac** – Patient with a primary diagnosis of medical illness at the time of the event that is not cardiovascular. • **Surgical-Cardiac** – Patient who is post-operative following cardiac surgery at the time of the event. • **Surgical-Noncardiac** – Patient who is pre-operative or post-operative with a surgical illness as the primary diagnosis that is not cardiac surgery at the time of the event. • **Trauma** – Patient with single or multiple traumas (significant injury to the head, chest, abdomen, kidney, urinary system, pelvis or spine, or upper or lower limb) as the primary diagnosis at the time of the event. • **NOTE** – Near-drowning is NOT a trauma.	○ Medical cardiac<br>○ Medical non-cardiac<br>○ Surgical cardiac<br>○ Surgical non-cardiac<br>○ Trauma
3.7	Immediate cause(s) of CPR event: (select all that apply)<br>Select primary cause resulting in cardiac arrest.	☐ ALTE/SIDS<br>☐ Airway obstruction or displacement<br>☐ Arrhythmia<br>☐ Drowning<br>☐ Electrolyte abnormality<br>☐ Elevated ICP<br>☐ Hypotension/shock<br>☐ Ingestion/Toxin<br>☐ Other respiratory failure (e.g. hypoxic)<br>☐ Seizures<br>☐ Trauma<br>☐ Other<br>☐ Unknown / Not documented
3.7.1	***If 'Immediate cause(s) of CPR event: (select all that apply)' is equal to 'Trauma' answer this question:***<br>Trauma Type<br>Select "Non-accidental" in cases where abuse or an assault is documented or the suspected cause of the cardiac arrest. Near drowning is NOT a Trauma. If the etiology of the trauma is not documented or unknown, select "Unknown".	○ Accidental<br>○ Non-accidental<br>○ Unknown / Not documented
3.7.2	***If 'Immediate cause(s) of CPR event: (select all that apply)' is equal to 'Other' answer this question:***<br>Immediate cause - Other Specify<br>Specify immediate other cause(s) of the CPR event.	
3.8	Relationship between Medical History and Cause of Arrest<br>Enter the most appropriate option. • **Medical history and cause of arrest unrelated** – Patient did not have any medical history or medical history and event cause are clearly unrelated (for example: trauma is the arrest cause and medical history of renal insufficiency). • **Medical history was the cause of arrest** – Patient diagnosed with the disease that is the cause of arrest (for example: arrest cause is long-QT syndrome and patient had been diagnosed prior to arrest) . • **Medical history could have been cause of arrest** – Patient who is diagnosed with a disease and it could have been the arrest cause (for example: patient is diagnosed with a primary arrythmogenic disorder prior to arrest and arrest rhythm was shockable but the rhythm strip is not available) • **Unknown if related** – Patient has a medical history but the arrest cause is unknown or it is unclear whether medical history and arrest cause are related.	○ Medical history (also if no medical history) and cause of arrest are unrelated<br>○ Medical history was the cause of arrest<br>○ Medical history could have been cause of arrest<br>○ Unknown if related

4. CPR Event Data - CPR Event Data

Number	Question	Answers
4.1	In-Hospital Cardiac Arrest or Out-Hospital Cardiac<br>**Select In Hospital** if compressions began while the patient was in the hospital setting (e.g., in patient floor, emergency department, operating room). Complete Step In-Hospital Cardiac Arrest. **Select Out of Hospital** if compressions began out of hospital, including en-route to hospital, and/or prior to hospital arrival. Select this option even if compressions continued once the patient arrived in the hospital. Complete Step Out-Hospital Cardiac Arrest.	○ In-Hospital (Complete Step In-Hospital Cardiac Arrest)<br>○ Out-Hospital (Complete Step Out-Hospital Cardiac Arrest)
4.2	Switch cpr_hosp_cardiac_arrest: 1, 2 or -1 (empty)	

5. CPR Event Data - In-Hospital Cardiac Arrest

Number	Question	Answers
	Step is not applicable. Select In-Hospital Cardiac Arrest in step CPR Event Data to enable this step.	
5.1	***If 'In-Hospital Cardiac Arrest or Out-Hospital Cardiac' is equal to 'In-Hospital (Complete Step In-Hospital Cardiac Arrest)' answer this question:***<br>Date of arrest (Demographics and Patient Characteristics)	
5.2	***If 'In-Hospital Cardiac Arrest or Out-Hospital Cardiac' is equal to 'In-Hospital (Complete Step In-Hospital Cardiac Arrest)' answer this question:***<br>Date and Time of Arrest<br>Enter the earliest time that the need for chest compressions (or defibrillation) was first recognized by telemetry or direct observation. ***If time is unknown, enter 00:00***.	_ _ _ *(dd-mm-yyyy)*<br>_ : _ *(hh:mm)*
5.3	***If 'In-Hospital Cardiac Arrest or Out-Hospital Cardiac' is equal to 'In-Hospital (Complete Step In-Hospital Cardiac Arrest)' answer this question:***<br>Location of Event<br>Select where the resuscitation occurred. If "Other", describe location below. Note: CICU is a cardiac intensive care unit; PACU is a post-anesthesia care unit for sedated post-surgical patients still under the care of an anesthesiologist.	○ ED<br>○ PICU<br>○ NICU<br>○ Operating room or PACU<br>○ Other (e.g. Medium care)
5.3.1	***If 'Location of Event' is equal to 'Other (e.g. Medium care)' answer this question:***<br>Location of Event - Other Specify<br>Specify Other Event Location.	___
5.4	***If 'In-Hospital Cardiac Arrest or Out-Hospital Cardiac' is equal to 'In-Hospital (Complete Step In-Hospital Cardiac Arrest)' answer this question:***<br>Time Chest Compressions Started<br>Enter the time that ***chest compressions began*** either by or CPR monitoring if pressure sensor pads were on for the first chest compression, or by direct observation. ***If time is unknown, enter 00:00***.	_ : _ *(hh:mm)*
5.5	***If 'In-Hospital Cardiac Arrest or Out-Hospital Cardiac' is equal to 'In-Hospital (Complete Step In-Hospital Cardiac Arrest)' answer this question:***<br>Were any interventions ALREADY IN PLACE at the time of event?	○ Yes<br>○ No
5.5.1	***If 'Were any interventions ALREADY IN PLACE at the time of event?' is equal to 'Yes' answer this question:***<br>If YES; please indicate Interventions ALREADY IN PLACE at time of event (first CCs) - select all that apply	☐ Assisted or mechanical ventilation (includes CPAP/BiPAP)<br>☐ Conscious/procedural sedation<br>☐ Dialysis/extracorporeal filtration therapy (ongoing)<br>☐ ECG<br>☐ End Tidal CO2 Monitoring<br>☐ Extracorporeal membrane oxygenation (ECMO)<br>☐ Implantable Cardiac defibrillator (ICD)<br>☐ Intra-arterial catheter<br>☐ Invasive airway/ETT<br>☐ IV/IO continuous infusion of antiarrhythmic(s)<br>☐ Pulse Oximetry<br>☐ Supplemental oxygen (e.g. nasal canule or high nasal flow)<br>☐ Other
5.5.1.1	***If 'If YES; please indicate Interventions ALREADY IN PLACE at time of event (first CCs) - select all that apply' is equal to 'Extracorporeal membrane oxygenation (ECMO)' answer this question:***<br>Confirm if patient was dependent on ECMO flow prior to compressions<br>Note: If patient is dependent on ECMO flow immediately prior to/at time of compressions, patient is NOT eligible for enrollment! Examples of ineligibility:<br>• Patient on ECMO, air in circuit; compressions initiated while air removed from circuit; patient resumes ECMO support<br>• Patient on ECMO; spontaneous decannulation; compressions initiated until re-cannulated; patient resumes ECMO support	○ NO, patient was NOT dependent on ECMO flow (eligible for enrollment; continue with data entry)<br>○ YES, patient was dependent on ECMO flow (not eligible; stop data entry and contact EMC team to delete this entry)
5.5.1.2	***If 'If YES; please indicate Interventions ALREADY IN PLACE at time of event (first CCs) - select all that apply' is equal to 'Other' answer this question:***<br>Indicate other Interventions in place at time of event	___
5.6	***If 'In-Hospital Cardiac Arrest or Out-Hospital Cardiac' is equal to 'In-Hospital (Complete Step In-Hospital Cardiac Arrest)' answer this question:***<br>Vascular Access - already in place when the need for chest compressions was first recognized.<br>**Yes** - If any of the following are already in place when the need for chest compressions and/or defibrillation was first recognized: • Peripheral Vein • Central Vein • Intraosseus (IO) • Umbilical vein (UVC) • Umbilical Artery (UAC). **Select "No"**, if none of these are in place prior to the need for chest compressions and/or defibrillation. **Select "Not Documented"** if not explicitly stated in the patient's chart.	○ Yes<br>○ No<br>○ Unknown / Not documented
5.7	***If 'In-Hospital Cardiac Arrest or Out-Hospital Cardiac' is equal to 'In-Hospital (Complete Step In-Hospital Cardiac Arrest)' answer this question:***<br>Vasoactive Agent - already in place when the need for chest compressions was first recognized.<br>**Yes** - If any of the following were already in place when the need for chest compressions and/or defibrillation was first recognized. **Select "No"** if no vasoactive agent was already in place when the need for chest compressions and/or defibrillation was first recognized or if started after the need for chest compressions and/or defibrillation. **Select "Not Documented"** if not explicitly stated in the patient's chart.	○ Yes<br>○ No<br>○ Unknown / Not documented
5.8	***If 'In-Hospital Cardiac Arrest or Out-Hospital Cardiac' is equal to 'In-Hospital (Complete Step In-Hospital Cardiac Arrest)' answer this question:***<br>Choose the selection that best describes this event	○ Patient was PULSELESS when the need for chest compressions was first recognized.<br>○ Patient initially had a pulse/heart rate (poor perfusion) requiring chest compressions PRIOR to becoming pulseless.<br>○ Patient had a pulse/heart rate (poor perfusion) requiring chest compressions, but did NOT become pulseless at any time during this event.
5.9	Switch best_describes_event = 2 or 3	
5.10	Switch best_describes_event = 1 or 2	

5.9.1	***If 'Switch best_describes_event = 2 or 3' is equal to '1' answer this question:***<br>Rhythm when patient WITH A PULSE first received compressions during the event<br>If patient received compressions with a pulse present, select the initial rhythm when the patient FIRST received chest compressions. **Select "Not Documented"** if not explicitly stated in the patient's chart.	◯ Bradycardia (<60/min)<br>◯ Pacemaker<br>◯ Sinus (including sinus tachycardia)<br>◯ Supraventricular tachyarrhythmia (SVTarrhy)<br>◯ Ventricular Tachycardia (VT) with a pulse<br>◯ Unknown / Not documented
5.10.1	***If 'Switch best_describes_event = 1 or 2' is equal to '1' answer this question:***<br>First Documented Pulseless Rhythm-- If the patient became pulseless at any time during the resuscitation, select the first pulseless rhythm identified. For the unmonitored patient, select the first rhythm identified when monitor was applied. Notes for Abstraction: • Enter the first (initial) cardiac rhythm recorded during the cardiac arrest event. • The initial rhythm can be obtained from a cardiac monitor, automated external defibrillator strip, or recorded on the code sheet. Select Unknown/Not Documented if there is no documentation of the first pulseless rhythm. • If there is conflicting information documented, e.g. the first pulseless rhythm documented on the code sheet is different than the first rhythm on the monitor strip, enter the first pulseless rhythm on the monitor strip. • The monitor strip is the preferred data source for this data element.	◯ Asystole<br>◯ Pulseless Electrical Activity (PEA)<br>◯ Ventricular fibrillation<br>◯ Ventricular tachycardia<br>◯ Other<br>◯ Unknown / Not documented
5.10.1.1	***If 'First Documented Pulseless Rhythm-- If the patient became pulseless at any time during the resuscitation, select the first pulseless rhythm identified. For the unmonitored patient, select the first rhythm identified when monitor was applied.' is equal to 'Other' answer this question:***<br>First Document Pulseless Rhythm Other Specify	
5.11	***If 'In-Hospital Cardiac Arrest or Out-Hospital Cardiac' is equal to 'In-Hospital (Complete Step In-Hospital Cardiac Arrest)' answer this question:***<br>Was defibrillation attempted?<br>**Yes** - If there was an attempt to deliver joules or energy via defibrillator or AED regardless of resulting rhythm.	◯ Yes<br>◯ No
5.11.1	***If 'Was defibrillation attempted?' is equal to 'Yes' answer this question:***<br>Number of Shocks<br>Enter the total number of shocks administered to the patient during the CPR event.	
5.11.2	***If 'Was defibrillation attempted?' is equal to 'Yes' answer this question:***<br>At what joule dosage were shocks delivered?<br>If shocks were delivered select whether joule dosage was only 4J/Kg or if (some) shocks were delivered at a different dossage.	◯ Only shocks 4J/Kg were delivered<br>◯ Shocks with other joule dosage were delivered
5.11.2.1	***If 'At what joule dosage were shocks delivered?' is equal to 'Shocks with other joule dosage were delivered' answer this question:***<br>Enter the number of shocks and at what joule dosage that differed from 4J/Kg.	
5.12	***If 'In-Hospital Cardiac Arrest or Out-Hospital Cardiac' is equal to 'In-Hospital (Complete Step In-Hospital Cardiac Arrest)' answer this question:***<br>Medications administered DURING the CPR event (select all that apply)<br>Select all medications from list that were administered from time compressions started and/or defibrillation was first recognized, to time of ROSC or death. If "Other" is selected, list other medications below.	☐ None<br>☐ Amiodarone<br>☐ Atropine<br>☐ Calcium<br>☐ Epinephrine - 1 dose only<br>☐ Epinephrine - 2-4 doses<br>☐ Epinephrine 5+ doses<br>☐ Fluid bolus<br>☐ Inhaled nitric oxide<br>☐ Lidocaine<br>☐ Magnesium Sulfate<br>☐ Other Vasopressors<br>☐ Sodium bicarbonate<br>☐ Vasopressin<br>☐ Glucose<br>☐ Other
5.12.1	***If 'Medications administered DURING the CPR event (select all that apply)' is equal to 'Other' answer this question:***<br>Medications administered - Other Specify	
5.13	***If 'In-Hospital Cardiac Arrest or Out-Hospital Cardiac' is equal to 'In-Hospital (Complete Step In-Hospital Cardiac Arrest)' answer this question:***<br>Non-Drug Interventions DURING ARREST<br>Select all that apply from list. If "Other" is selected, describe the intervention below. Note: ECPR is defined as a transfer to ECMO during the resuscitation event or before attaining sustained ROSC (>20 minutes).	☐ None<br>☐ Endotracheal intubation<br>☐ Echocardiogram (intra-arrest)<br>☐ Cardiopulmonary bypass/ extracorporeal CPR (ECPR)<br>☐ Chest tube(s) inserted<br>☐ Needle thoracostomy<br>☐ Pacemaker, transcutaneous<br>☐ Pacemaker, transvenous or epicardial<br>☐ Pericardiocentesis<br>☐ Other Non-Drug Interventions
5.13.1	***If 'Non-Drug Interventions DURING ARREST' is equal to 'Other Non-Drug Interventions' answer this question:***<br>Non-Drug Interventions - Other	
5.13.2	***If 'Non-Drug Interventions DURING ARREST' is equal to 'Cardiopulmonary bypass/ extracorporeal CPR (ECPR)' answer this question:***<br>If placed on ECMO after initiation of CCs (eCPR), indicate final cannulation site<br>Indicate anatomic location of final cannulation site, even if immediate previous attempts were elsewhere.	◯ Neck<br>◯ Chest<br>◯ Groin<br>◯ Other
5.13.2.1	***If 'If placed on ECMO after initiation of CCs (eCPR), indicate final cannulation site' is equal to 'Other' answer this question:***<br>Other cannulation site	

5.14	***If 'In-Hospital Cardiac Arrest or Out-Hospital Cardiac' is equal to 'In-Hospital (Complete Step In-Hospital Cardiac Arrest)' answer this question:***<br>Was physiologic monitoring used as the primary guide for quality of compressions? Example: ETCO2 or Art line was used to guide depth of CCs instead of Defib/CC monitor.<br>Select **Yes** if the code leader used the physiologic monitoring as a guide for the depth of compressions. This may occur when the depth of compressions (via defibrillator/CC monitor feedback) is not resulting in optimal hemodynamic responses (via bedside monitor – arterial, central venous, ETCO2 readings), as determined and instructed by code leader. **Select "Unsure"** if not explicitly stated in the patient's chart or unable to determine.	○ Yes<br>○ No<br>○ Unsure if used, but available
5.14.1	***If 'Was physiologic monitoring used as the primary guide for quality of compressions? Example: ETCO2 or Art line was used to guide depth of CCs instead of Defib/CC monitor.' is not equal to 'No' answer this question:***<br>Physiologic monitoring available<br>Select ALL options that were used during the CPR event. If "Other" is selected, please describe below.	☐ Arterial line<br>☐ CVP<br>☐ EEG<br>☐ NIRS<br>☐ ETCO2 - via bedside<br>☐ ETCO2 - via defibrillator/CPR monitor (Zoll, Philips, etc.)<br>☐ Compression sensor pad<br>☐ Unknown / Not documented<br>☐ Other
5.14.1.1	***If 'Physiologic monitoring available' is equal to 'Other' answer this question:***<br>Physiologic monitoring available - Other Specify	
5.15	***If 'In-Hospital Cardiac Arrest or Out-Hospital Cardiac' is equal to 'In-Hospital (Complete Step In-Hospital Cardiac Arrest)' answer this question:***<br>Reason Resuscitation Ended<br>**"ROSC"**: Patient attained ROSC (Return of Spontaneous Circulation) for more than 20 minutes and no longer required chest compressions. Enter the date and time that sustained ROSC began. (DD/MM/YYYY, HH:MM, military time). **"ROC with ECMO"**: Patient was transferred to ECMO during the resuscitation event and no longer required chest compressions. Enter the date and time that sustained ROC began. (DD/MM/YYYY, HH:MM, military time). **"Died – Efforts Terminated"**: Patient had no Return of Spontaneous Circulation; did not respond to Advanced Life Support (ALS), unable to achieve sustained ROC, or there was an advance directive limiting ALS, or there were restrictions placed by the family of the patient during the event (i.e., family requested event be terminated). Enter the date and time of death as recorded in the patient's chart. (DD/MM/YYYY, HH:MM, military time).	○ ROSC<br>○ Died - Efforts terminated, no sustained ROSC<br>○ ROC with ECMO
5.16	***If 'In-Hospital Cardiac Arrest or Out-Hospital Cardiac' is equal to 'In-Hospital (Complete Step In-Hospital Cardiac Arrest)' answer this question:***<br>Date and time of arrest (CPR Event Data)	
5.17	***If 'In-Hospital Cardiac Arrest or Out-Hospital Cardiac' is equal to 'In-Hospital (Complete Step In-Hospital Cardiac Arrest)' answer this question:***<br>Date and Time Sustained ROC began (lasting >20 min) or Resuscitation efforts were terminated (end of event)<br>Indicates the end of this CPR event. ***If time is unknown, enter 00:00***	*(dd-mm-yyyy)*<br>*(hh:mm)*
5.18	***If 'In-Hospital Cardiac Arrest or Out-Hospital Cardiac' is equal to 'In-Hospital (Complete Step In-Hospital Cardiac Arrest)' answer this question:***<br>Total Duration of CPR for this event (autocalculated)	
5.19	***If 'In-Hospital Cardiac Arrest or Out-Hospital Cardiac' is equal to 'In-Hospital (Complete Step In-Hospital Cardiac Arrest)' answer this question:***<br>Total Duration of CPR for this event (min). If unsure, provide an educated estimate rounded UP to the nearest 5 minute interval.<br>Enter the number of minutes that CPR was performed during this resuscitation. Use CPR monitoring devices, direct observation, code sheets to determine the total time. If the patient regained circulation (ROSC, Return of Spontaneous Circulation) for less than 20 minutes and required more CPR, add the time of each CPR epoch. The event ends once the patient has attained ROSC, return of sustained circulation for more than 20 minutes. Add the total time of chest compressions from the first chest compression to the beginning of sustained ROSC (>20 minutes). If unsure, provide an educated estimate rounded UP to the nearest 5-minute interval.	minute(s)
5.19.1	***If 'Total Duration of CPR for this event (min). If unsure, provide an educated estimate rounded UP to the nearest 5 minute interval.' is greater or equal than '1' answer this question:***<br>Duration Source. Indicate how duration of CPR was calculated.<br>Indicate if the duration of CPR was calculated per available clinical documentation or whether it was an educated estimate (documentation unclear or unavailable).	○ Duration indicated is per clinical documentation<br>○ Duration indicated is an educated estimate
5.20	***If 'In-Hospital Cardiac Arrest or Out-Hospital Cardiac' is equal to 'In-Hospital (Complete Step In-Hospital Cardiac Arrest)' answer this question:***<br>In-Hospital CPR Event Comments<br>Do not lead entry with a "-" [dash] (will export as an error).	
	**Site Data Files** - Upload any pertinent files to THIS section (anonymized).	
5.21	***If 'In-Hospital Cardiac Arrest or Out-Hospital Cardiac' is equal to 'In-Hospital (Complete Step In-Hospital Cardiac Arrest)' answer this question:***<br>Site Data Files upload<br>Select Yes to upload Site Data Files.	○ Yes<br>○ No
5.21.1	***If 'Site Data Files upload' is equal to 'Yes' answer this question:***<br>CPR Quality Monitoring Device File (defibrillator file)	
5.21.2	***If 'Site Data Files upload' is equal to 'Yes' answer this question:***<br>Bedside monitor printouts<br>Instructions for how to download monitor printouts can be found in REDCap bookmarks (left) or the File Repository.<br>Download at page speed of 12.5mm/sec.	

5.21.3	***If 'Site Data Files upload' is equal to 'Yes' answer this question:***<br>Indicate: 1) data that are within this file/document upload and 2) format of the data file	☐ Arterial pressures<br>☐ Central venous pressures<br>☐ ECG<br>☐ Echo Report<br>☐ EEG<br>☐ ETCO2<br>☐ NIRS<br>☐ SPO2<br>☐ Other - please specify<br>☐ Indicate data format: waveforms (pdf)<br>☐ Indicate data format: numeric (xls, csv, xml)
5.21.3.1	***If 'Indicate: 1) data that are within this file/document upload and 2) format of the data file' is equal to 'Other - please specify' answer this question:***<br>Specify other data not listed above	
5.21.4	***If 'Site Data Files upload' is equal to 'Yes' answer this question:***<br>NIRS Data	
5.21.5	***If 'Site Data Files upload' is equal to 'Yes' answer this question:***<br>Other File 1<br>Indicate contents below.	
5.21.6	***If 'Site Data Files upload' is equal to 'Yes' answer this question:***<br>Indicate: 1) data that are within this file/document upload and 2) format of the data file	☐ Arterial pressures<br>☐ Central venous pressures<br>☐ ECG<br>☐ Echo Report<br>☐ EEG<br>☐ ETCO2<br>☐ NIRS<br>☐ SPO2<br>☐ Other - please specify<br>☐ Indicate data format: waveforms (pdf)<br>☐ Indicate data format: numeric (xls, csv, xml)
5.21.6.1	***If 'Indicate: 1) data that are within this file/document upload and 2) format of the data file' is equal to 'Other - please specify' answer this question:***<br>Other contents of File 1<br>Contents not listed above.	
5.21.7	***If 'Site Data Files upload' is equal to 'Yes' answer this question:***<br>Other File 2<br>Indicate contents below.	
5.21.8	***If 'Site Data Files upload' is equal to 'Yes' answer this question:***<br>Indicate: 1) data that are within this file/document upload and 2) format of the data file	☐ Arterial pressures<br>☐ Central venous pressures<br>☐ ECG<br>☐ Echo Report<br>☐ EEG<br>☐ ETCO2<br>☐ NIRS<br>☐ SPO2<br>☐ Other - please specify<br>☐ Indicate data format: waveforms (pdf)<br>☐ Indicate data format: numeric (xls, csv, xml)
5.21.8.1	***If 'Indicate: 1) data that are within this file/document upload and 2) format of the data file' is equal to 'Other - please specify' answer this question:***<br>Other contents of File 2<br>Contents not listed above.	
5.21.9	***If 'Site Data Files upload' is equal to 'Yes' answer this question:***<br>Other File 3<br>Indicate contents below.	
5.21.10	***If 'Site Data Files upload' is equal to 'Yes' answer this question:***<br>Indicate: 1) data that are within this file/document upload and 2) format of the data file	☐ Arterial pressures<br>☐ Central venous pressures<br>☐ ECG<br>☐ Echo Report<br>☐ EEG<br>☐ ETCO2<br>☐ NIRS<br>☐ SPO2<br>☐ Other - please specify<br>☐ Indicate data format: waveforms (pdf)<br>☐ Indicate data format: numeric (xls, csv, xml)
5.21.10.1	***If 'Indicate: 1) data that are within this file/document upload and 2) format of the data file' is equal to 'Other - please specify' answer this question:***<br>Other contents of File 3<br>Contents not listed above.	

6. CPR Event Data - Out-Hospital Cardiac Arrest

Number	Question	Answers
	Step is not applicable. Select Out-Hospital Cardiac Arrest in step CPR Event Data to enable this step.	
6.1	***If 'In-Hospital Cardiac Arrest or Out-Hospital Cardiac' is equal to 'Out-Hospital (Complete Step Out-Hospital Cardiac Arrest)' answer this question:***<br>Date of arrest (Demographics and Patient Characteristics)	
6.2	***If 'In-Hospital Cardiac Arrest or Out-Hospital Cardiac' is equal to 'Out-Hospital (Complete Step Out-Hospital Cardiac Arrest)' answer this question:***<br>Date and Time of Arrest<br>Earliest time that the need for chest compressions was first recognized by direct observation. ***If time is unknown, enter 00:00***.	*(dd-mm-yyyy)*<br>: *(hh:mm)*
6.3	***If 'In-Hospital Cardiac Arrest or Out-Hospital Cardiac' is equal to 'Out-Hospital (Complete Step Out-Hospital Cardiac Arrest)' answer this question:***<br>Location of Event<br>Select where the resuscitation occurred. If "Other", describe location below.	◯ Home/Private space<br>◯ Public space<br>◯ Other
6.3.1	***If 'Location of Event' is equal to 'Other' answer this question:***<br>Location of Event - Other Specify<br>Specify Other Event Location.	
6.4	***If 'In-Hospital Cardiac Arrest or Out-Hospital Cardiac' is equal to 'Out-Hospital (Complete Step Out-Hospital Cardiac Arrest)' answer this question:***<br>Witnessed Arrest<br>**Yes** - If the onset of the cardiopulmonary arrest was directly observed by someone (family, lay bystander, employee, or health care professional).	◯ Yes<br>◯ No<br>◯ Unknown/Not documented
6.5	***If 'In-Hospital Cardiac Arrest or Out-Hospital Cardiac' is equal to 'Out-Hospital (Complete Step Out-Hospital Cardiac Arrest)' answer this question:***<br>Time Chest Compressions Started<br>Enter the time that ***chest compressions began*** either by or CPR monitoring if pressure sensor pads were on for the first chest compression, or by direct observation. ***If time is unknown, enter 00:00***.	: *(hh:mm)*
6.6	***If 'In-Hospital Cardiac Arrest or Out-Hospital Cardiac' is equal to 'Out-Hospital (Complete Step Out-Hospital Cardiac Arrest)' answer this question:***<br>Bystander CPR<br>**Yes** - If a lay person performed CPR prior to EMS arrival or transport to the hospital. Select "Unknown/Not Documented" if this is not explicitly stated or documented.	◯ Yes<br>◯ No<br>◯ Unknown / Not documented
6.7	***If 'In-Hospital Cardiac Arrest or Out-Hospital Cardiac' is equal to 'Out-Hospital (Complete Step Out-Hospital Cardiac Arrest)' answer this question:***<br>Was an AED attached to the patient?<br>Yes - If an Automated External Defibrillator (AED) was used either by a civilian, text message system alerted lay person or police/fire fighter in the field. 'Use' in this question means attached to the patient and is independent whether a shock was advised or not. Select "Unknown/Not Documented" if this is not explicitly stated or documented.	◯ Yes<br>◯ No<br>◯ Unknown/Not Documented
6.7.1	***If 'Was an AED attached to the patient?' is equal to 'Yes' answer this question:***<br>Who provided the AED?<br>Select who provided the AED. If unsure or unknown select this option AS WELL, other people can still be selected for example if it is certain that EMS provided CPR but uncertain if a parent provided CPR prior to EMS arriving. 'AED use' in this question means attached to the patient and is independent whether a shock was advised or not.	☐ Parent/Caregiver<br>☐ Bystander<br>☐ Message alerted lay-rescuer<br>☐ Police<br>☐ Firefighter<br>☐ Unsure/Unknown
6.8	***If 'In-Hospital Cardiac Arrest or Out-Hospital Cardiac' is equal to 'Out-Hospital (Complete Step Out-Hospital Cardiac Arrest)' answer this question:***<br>Time EMS arrived at scene<br>Enter the time that the first EMS (ambulance or MMT/HEMS) arrived. (HH:MM, military time). If no bystander BLS was given, time of EMS arrival can occur before time chest compressions started. ***If time is unknown, enter 00:00***	: *(hh:mm)*
6.9	***If 'In-Hospital Cardiac Arrest or Out-Hospital Cardiac' is equal to 'Out-Hospital (Complete Step Out-Hospital Cardiac Arrest)' answer this question:***<br>Who performed CPR?<br>Select all people that performed CPR. If unsure or unknown select this option AS WELL, other people can still be selected for example if it is certain that EMS provided CPR but uncertain if a parent provided CPR prior to EMS arriving.	☐ Parent/Caregiver<br>☐ Bystander<br>☐ Message alerted lay-rescuer<br>☐ Police<br>☐ Firefigher<br>☐ EMS<br>☐ Unsure/Unknown
6.10	***If 'In-Hospital Cardiac Arrest or Out-Hospital Cardiac' is equal to 'Out-Hospital (Complete Step Out-Hospital Cardiac Arrest)' answer this question:***<br>Was defibrillation attempted?<br>**Yes** - If there was an attempt to deliver joules or energy via defibrillator or AED regardless of resulting rhythm.	◯ Yes<br>◯ No
6.10.1	***If 'Was defibrillation attempted?' is equal to 'Yes' answer this question:***<br>Number of Shocks<br>Enter the number of shocks (AED and defibrillator combined) administered to the patient during the resuscitation. **If the number of shocks was unknown or not documented, enter '98'.**	
6.10.2	***If 'Was defibrillation attempted?' is equal to 'Yes' answer this question:***<br>At what joule dosage were shocks delivered?<br>If shocks were delivered select whether joule dosage was only 4J/Kg or if (some) shocks were delivered at a different dossage.	◯ Only shocks 4J/Kg were delivered<br>◯ Shocks with other joule dosage were delivered
6.10.2.1	***If 'At what joule dosage were shocks delivered?' is equal to 'Shocks with other joule dosage were delivered' answer this question:***<br>Enter the number of shocks and at what joule dosage that differed from 4J/Kg.	

6.11	***If 'In-Hospital Cardiac Arrest or Out-Hospital Cardiac' is equal to 'Out-Hospital (Complete Step Out-Hospital Cardiac Arrest)' answer this question:***<br>Initial rhythm when compressions started<br>Select the rhythm when compressions first started. If the patient is un-monitored, select the first rhythm when monitor became available. • Select "Ventricular fibrillation" if an AED was attached to the patient and a shock performed prior to the first official rhythm check also if no rhythm strip is available. • Select "Unknown" if no rhythm was ever documented during the CPR event. • If "Other" is selected, describe the rhythm below. Note: *This list combines both pulseless and pulsed rhythms. This is different from Step In-Hospital Cardiac Arrest, please review carefully.*	○ Asystole<br>○ Pulseless Electrical Activity (PEA)<br>○ Ventricular fibrillation<br>○ Pulseless Ventricular tachycardia<br>○ Bradycardia<br>○ Accelerated idioventricular rhythm (AIVR)<br>○ Pacemaker<br>○ Sinus (including Sinus Tachycardia)<br>○ Supraventricular tachyarrhythmia (SVTarrhy)<br>○ Ventricular Tachycardia (VT) with a pulse<br>○ Other<br>○ Unknown / Not documented
6.11.1	***If 'Initial rhythm when compressions started' is equal to 'Other' answer this question:***<br>Initial Rhythm - Other<br>Specify Other Initial Rhythm.	
6.12	***If 'In-Hospital Cardiac Arrest or Out-Hospital Cardiac' is equal to 'Out-Hospital (Complete Step Out-Hospital Cardiac Arrest)' answer this question:***<br>What was the source of the initial rhythm?<br>If the rhythm strip is available please upload in the Site Data Files section.	○ AED Shock (no ECG available)<br>○ AED ECG<br>○ Defibrillator ECG<br>○ EMS monitor<br>○ Electronic patient file information, source not specified<br>○ Unknown/Not documented
6.12.1	***If 'What was the source of the initial rhythm?' is not equal to 'Unknown/Not documented' answer this question:***<br>Rhythm strip available for upload?<br>Is the original rhythm strip with the initial rhythm available? If so, please upload in the Site Data Files section below.	○ Yes<br>○ No
6.13	***If 'In-Hospital Cardiac Arrest or Out-Hospital Cardiac' is equal to 'Out-Hospital (Complete Step Out-Hospital Cardiac Arrest)' answer this question:***<br>Time EMS leaving the scene<br>Enter the time that EMS treated the patient at the scene (i.e. time of leaving the scene with the patient/treatment interval) (HH:MM, military time). ***If time is unknown, enter 00:00***	: *(hh:mm)*
6.14	***If 'In-Hospital Cardiac Arrest or Out-Hospital Cardiac' is equal to 'Out-Hospital (Complete Step Out-Hospital Cardiac Arrest)' answer this question:***<br>Time EMS arrived at hospital<br>Enter the date and time that EMS arrived at the hospital (HH:MM, military time). ***If time is unknown, enter 00:00***	: *(hh:mm)*
6.15	***If 'In-Hospital Cardiac Arrest or Out-Hospital Cardiac' is equal to 'Out-Hospital (Complete Step Out-Hospital Cardiac Arrest)' answer this question:***<br>Medications administered during CPR event (check all that apply)<br>Select all medications that were administered from time the need compressions and/or defibrillation was first recognized, to time of ROC or death. If "Other" is selected, list other medications below.	☐ None<br>☐ Amiodarone<br>☐ Atropine<br>☐ Calcium<br>☐ Epinephrine - 1 dose only<br>☐ Epinephrine - 2-4 doses<br>☐ Epinephrine - 5+ doses<br>☐ Fluid bolus<br>☐ Lidocaine<br>☐ Magnesium Sulfate<br>☐ Sodium bicarbonate<br>☐ Vasopressin<br>☐ Glucose<br>☐ Other
6.15.1	***If 'Medications administered during CPR event (check all that apply)' is equal to 'Other' answer this question:***<br>Medications administered during CPR event - Other<br>Specify Other Medications administered during CPR event.	
6.16	***If 'In-Hospital Cardiac Arrest or Out-Hospital Cardiac' is equal to 'Out-Hospital (Complete Step Out-Hospital Cardiac Arrest)' answer this question:***<br>Non-Drug Interventions<br>Select all that apply. If "Other" is selected, describe the intervention below.	☐ None<br>☐ Endotracheal intubation<br>☐ Echocardiogram (intra-arrest)<br>☐ Cardiopulmonary bypass/ extracorporeal CPR (eCPR)<br>☐ Chest tube(s) inserted<br>☐ Needle thoracostomy<br>☐ Pacemaker, transcutaneious<br>☐ Pacemaker, transvenous or epicardial<br>☐ Pericardiocentesis<br>☐ Other Non-Drug Interventions (e.g. intraosseous infusion)
6.16.1	***If 'Non-Drug Interventions' is equal to 'Other Non-Drug Interventions (e.g. intraosseous infusion)' answer this question:***<br>Non-Drug Interventions - Other<br>Specify Other Non-Drug Interventions.	
6.16.2	***If 'Non-Drug Interventions' is equal to 'Cardiopulmonary bypass/ extracorporeal CPR (eCPR)' answer this question:***<br>If placed on ECMO after initiation of CCs (eCPR), indicate final cannulation site<br>Final anatomic cannulation site, even if immediate previous attempts were elsewhere.	○ Neck<br>○ Chest<br>○ Groin<br>○ Other

6.16.2.1	***If 'If placed on ECMO after initiation of CCs (eCPR), indicate final cannulation site' is equal to 'Other' answer this question:***<br>Other cannulation site<br>Specify Other cannulation site.	
6.17	***If 'In-Hospital Cardiac Arrest or Out-Hospital Cardiac' is equal to 'Out-Hospital (Complete Step Out-Hospital Cardiac Arrest)' answer this question:***<br>Was physiologic monitoring used to guide compression depth during resuscitation? Example: Code leader was using ETCO2 as determination of adequate CC depth (not CC monitor/defib CC feedback).<br>Select **Yes** if the code leader used the physiologic monitoring as a guide for the depth of compressions. This may occur when the depth of compressions (via defibrillator/CC monitor feedback) is not resulting in optimal hemodynamic responses (via bedside monitor – arterial, central venous, ETCO2 readings), as determined and instructed by code leader. **Select "Unknown"** if not explicitly stated in the patient's chart or unable to determine.	○ Yes<br>○ No<br>○ Unknown / Not documented if used, but available
6.18	***If 'In-Hospital Cardiac Arrest or Out-Hospital Cardiac' is equal to 'Out-Hospital (Complete Step Out-Hospital Cardiac Arrest)' answer this question:***<br>Physiologic monitoring available<br>Select all options that were used during the CPR event. If "Other" is selected, please describe below.	☐ None<br>☐ ETCO2<br>☐ Compression sensor pad<br>☐ Arterial line<br>☐ Other
6.18.1	***If 'Physiologic monitoring available' is equal to 'Compression sensor pad' answer this question:***<br>Physiologic monitoring available - Other<br>Specify Other available Physiologic monitoring.	
6.19	***If 'In-Hospital Cardiac Arrest or Out-Hospital Cardiac' is equal to 'Out-Hospital (Complete Step Out-Hospital Cardiac Arrest)' answer this question:***<br>Reason Resuscitation Ended<br>**"ROSC"**: Patient attained ROSC (Return of Sustained Circulation) for more than 20 minutes and no longer required chest compressions. Enter the date and time that sustained ROSC began. (DD/MM/YYYY, HH:MM, military time). **"ROC with ECMO"**: Patient was transferred to ECMO during the resuscitation event and no longer required chest compressions. Enter the date and time that sustained ROC began. (DD/MM/YYYY, HH:MM, military time). **"Died – Efforts Terminated"**: Patient had no Return of Spontaneous Circulation; did not respond to Advanced Life Support (ALS), unable to achieve sustained ROC, or there was an advance directive limiting ALS, or there were restrictions placed by the family of the patient during the event (i.e., family requested event be terminated). Enter the date and time of death as recorded in the patient's chart. (DD/MM/YYYY, HH:MM, military time).	○ ROSC<br>○ Died - Efforts terminated, no sustained ROSC<br>○ ROC with ECMO
6.19.1	***If 'Reason Resuscitation Ended' is not equal to 'Died - Efforts terminated, no sustained ROSC' answer this question:***<br>ROSC Attained<br>If sustained ROSC was attained (lasting longer than 20 minutes), select where this occurred. Select the location of the patient at the beginning of sustained ROSC.	○ At Scene<br>○ En Route<br>○ After Arrival to Hospital<br>○ Unknown / Not Documented
6.20	***If 'In-Hospital Cardiac Arrest or Out-Hospital Cardiac' is equal to 'Out-Hospital (Complete Step Out-Hospital Cardiac Arrest)' answer this question:***<br>Date of arrest (Demographics and Patient Characteristics)	
6.21	***If 'In-Hospital Cardiac Arrest or Out-Hospital Cardiac' is equal to 'Out-Hospital (Complete Step Out-Hospital Cardiac Arrest)' answer this question:***<br>Date and Time Sustained ROC began (lasting >20 min) or Resuscitation efforts were terminated (end of event)<br>Indicates the end of this CPR event. Enter 00:00 for time if unknown.	*(dd-mm-yyyy)*<br>: *(hh:mm)*
6.22	***If 'In-Hospital Cardiac Arrest or Out-Hospital Cardiac' is equal to 'Out-Hospital (Complete Step Out-Hospital Cardiac Arrest)' answer this question:***<br>Total Duration of CPR for this event (autocalculated)	
6.23	***If 'In-Hospital Cardiac Arrest or Out-Hospital Cardiac' is equal to 'Out-Hospital (Complete Step Out-Hospital Cardiac Arrest)' answer this question:***<br>Total CPR duration: Total minutes of CPR for this event (min). If unsure, provide an educated estimate rounded UP to the nearest 5-minute interval.<br>Indicate total time of CCs from the first CC to the beginning of sustained ROSC (>20 minutes) or death. • If Yes was selected above, calculate total duration of CPR given during this resuscitation event. Should include both out of hospital and in hospital (if applicable) CPR time. Use direct observation, video monitoring, or CPR data to calculate the total duration of CPR. • If ROSC was attained for less than 20 minutes and chest compressions resumed, enter all epochs of chest compressions required during this event. • The event does not end until ROSC lasts >20 minutes. • If unsure, provide an educated estimate rounded UP to the nearest 5-minute interval.	minute(s)
6.23.1	***If 'Total CPR duration: Total minutes of CPR for this event (min). If unsure, provide an educated estimate rounded UP to the nearest 5-minute interval.' is greater or equal than '1' answer this question:***<br>Duration Source<br>Indicate if the duration of CPR was calculated per available clinical documentation or whether it was an educated estimate (documentation unclear or unavailable).	○ Duration indicated is per clinical documentation<br>○ Duration indicated is an educated estimate
6.24	***If 'In-Hospital Cardiac Arrest or Out-Hospital Cardiac' is equal to 'Out-Hospital (Complete Step Out-Hospital Cardiac Arrest)' answer this question:***<br>Out-of-Hospital CPR Event Comments<br>Do not lead entry with a "-" [dash] (will export as an error).	
	**Site Data Files** - Upload any pertinent files to THIS section (anonymized).	
6.25	***If 'In-Hospital Cardiac Arrest or Out-Hospital Cardiac' is equal to 'Out-Hospital (Complete Step Out-Hospital Cardiac Arrest)' answer this question:***<br>Site Data Files upload<br>Select Yes to upload Site Data Files.	○ Yes<br>○ No
6.25.1	***If 'Site Data Files upload' is equal to 'Yes' answer this question:***<br>CPR Quality Monitoring Device File (defibrillator file)	
6.25.2	***If 'Site Data Files upload' is equal to 'Yes' answer this question:***<br>Bedside monitor printouts<br>Instructions for how to download monitor printouts can be found in REDCap bookmarks (left) or the File Repository.<br>Download at page speed of 12.5mm/sec.	

6.25.3	***If 'Site Data Files upload' is equal to 'Yes' answer this question:***<br>Indicate: 1) data that are within this file/document upload and 2) format of the data file	☐ Arterial pressures<br>☐ Central venous pressures<br>☐ ECG<br>☐ Echo Report<br>☐ EEG<br>☐ ETCO2<br>☐ NIRS<br>☐ SPO2<br>☐ Other - please specify<br>☐ Indicate data format: waveforms (pdf)<br>☐ Indicate data format: numeric (xls, csv, xml)
6.25.3.1	***If 'Indicate: 1) data that are within this file/document upload and 2) format of the data file' is equal to 'Other - please specify' answer this question:***<br>Specify other data not listed above	
6.25.4	***If 'Site Data Files upload' is equal to 'Yes' answer this question:***<br>Other File 1<br>Indicate contents below.	
6.25.5	***If 'Site Data Files upload' is equal to 'Yes' answer this question:***<br>Other contents of File 1<br>Contents not listed above.	

7. Short Term Outcomes, Mortality and Unexplained Death - Outcomes

Number	Question	Answers
7.1	Survival to ICU Discharge<br>**Yes** - If the patient survived until they were discharged from the ICU. **No** - If the patient did not survive to ICU discharge or no ROC after OHCA and never admitted to the ICU. Continue on to the end of life questions.	○ Yes<br>○ No
7.1.1	***If 'Survival to ICU Discharge' is equal to 'Yes' answer this question:***<br>Date and time of ICU discharge<br>If patient survived to ICU discharge, enter date and time of discharge from intensive careunit. (DD/MM/YYYY, HH:MM, military time). **If time is unknown, enter 00:00.**	*(dd-mm-yyyy)*<br>*(hh:mm)*
7.1.2	***If 'Survival to ICU Discharge' is equal to 'Yes' answer this question:***<br>PICU Discharge Disposition<br>If patient was discharged alive from the ICU, select the option that best describes the patient's post-ICU discharge destination.	○ Transfer to other (academic) PICU<br>○ Medium care ward, same hospital<br>○ Medium care ward, secondary care hospital<br>○ Rehabilitation center<br>○ Home<br>○ Hospice - Home<br>○ Hospice - Health Care Facility<br>○ Other Healthcare Facility<br>○ Not documented or unknown
7.1.2.1	***If 'PICU Discharge Disposition' is equal to 'Other Healthcare Facility' answer this question:***<br>PICU Discharge Disposition - Other<br>Specify which hospital or which 'other healthcare facility'.	
7.2	Survival to hospital discharge?<br>**Yes** - If the patient survived to hospital discharge. **No** - If the patient did not survive to hospital discharge. Continue on to the end of life questions.	○ Yes<br>○ No
7.2.1	***If 'Survival to hospital discharge?' is equal to 'Yes' answer this question:***<br>Date and Time of Hospital Discharge<br>If the patient survived to hospital discharge, enter the time and date of discharge. (DD-MM-YYYY, HH:MM, military time). **If time is unknown, enter 00:00.**	*(dd-mm-yyyy)*<br>*(hh:mm)*
7.2.2	***If 'Survival to hospital discharge?' is equal to 'Yes' answer this question:***<br>Discharge Disposition<br>If patient was discharged alive from the hospital, select the option that best describes the patient's post-hospital discharge destination. This field refers to the discharge destination after the patient is discharged from the acute care hospital. Refer to the discharge reports and latest documentation for this information.	○ Home<br>○ Transfer to pediatric ward secondary care hospital<br>○ Transfer to other (academic) PICU<br>○ Rehabilitation center<br>○ Hospice - Home<br>○ Hospice - Health Care Facility<br>○ Other Healthcare Facility<br>○ Not Documented or Unable to Determine
7.2.2.1	***If 'Discharge Disposition' is equal to 'Other Healthcare Facility' answer this question:***<br>Hospital Discharge Disposition - Other<br>Specify which hospital or which 'other healthcare facility'.	
7.2.3	***If 'Survival to hospital discharge?' is equal to 'Yes' answer this question:***<br>Deceased after hospital discharge?<br>**Yes** - If the patient survived to hospital discharge but deceased after discharge. **No** - If the patient survived to date. Note: the answer to this question can, if new information is provided at a later stage, be altered.	○ Yes<br>○ No
7.3	Switch no icu discharge or no hospital discharge or deceased after discharge: 1=Yes, 0=No	
7.3.1	***If 'Switch no icu discharge or no hospital discharge or deceased after discharge: 1=Yes, 0=No' is equal to '1' answer this question:***<br>What was the cause of death?<br>What best describes the cause of death? Select all that apply. Withholding or withdrawing of life-sustaining therapy or life support is a process by which various medical interventions either are not given to or are taken away from patients with the expectation that they will die from their underlying illnesses.	☐ Discontinuation of CPR (primary arrest)<br>☐ Multi-organ failure<br>☐ Cardiorespiratory failure<br>☐ Re-arrest without ROC<br>☐ Brain death<br>☐ WLST due to neurologic prognosis<br>☐ WLST due to respiratory failure<br>☐ WLST due to cardiac failure<br>☐ Unknown/Not specified
7.3.2	***If 'Switch no icu discharge or no hospital discharge or deceased after discharge: 1=Yes, 0=No' is equal to '1' answer this question:***<br>Was there Donation after Death?<br>Select which criteria was met: **Yes** - Donation after Cardiac/Circulatory Death (DCD) (*irreversible cessation of circulatory and respiratory functions*). **Yes** - Donation after Brain Death (*irreversible cessation of all functions of the entire brain, including the brain stem*). **No** - Organs were not recovered.	○ No, organs not recovered<br>○ Yes, after Brain Death (spontaneous heart beat)<br>○ Yes, after Cardiac/Circulatory Death (brain intact; non-beating heart)
7.4	Declared DNAR during this admission?<br>DNAR, Do Not Attempt Resuscitation.	○ Yes<br>○ No
7.4.1	***If 'Declared DNAR during this admission?' is equal to 'Yes' answer this question:***<br>Date and Time of DNAR order<br>If yes, enter the date and time of DNAR order. (DD-MM-YYYY, HH:MM, military time). **If time is unknown, enter 00:00.**	*(dd-mm-yyyy)*<br>*(hh:mm)*
7.3.3	***If 'Switch no icu discharge or no hospital discharge or deceased after discharge: 1=Yes, 0=No' is equal to '1' answer this question:***<br>Date and Time of Death<br>Enter the date and time at which the patient died or was pronounced brain dead. (DD-MM-YYYY, HH:MM, military time). **If time is unknown, enter 00:00.**	*(dd-mm-yyyy)*<br>*(hh:mm)*

7.5	Switch out_cause_of_death = checked 6 or 7 or 8	
7.5.1	***If 'Switch out_cause_of_death = checked 6 or 7 or 8' is equal to '1' answer this question:***<br>On what basis was decided to withdraw life sustaining therapies?<br>Select all that apply. What were the investigations performed and/or meetings held that were used to support the decision to withdraw life sustaining therapies? Select "Unknown/Not documented" if not explicitly stated in thepatient's chart.	☐ Multidisciplinary meeting<br>☐ Neurologic examination<br>☐ EEG<br>☐ Ultrasound Brain<br>☐ CT Brain<br>☐ MRI Brain<br>☐ SSEP<br>☐ Cerebral biomarkers<br>☐ CPR variables (duration, lactate, pH etc.)<br>☐ Medical history<br>☐ Other<br>☐ Unknown/Not documented
7.5.1.1	***If 'On what basis was decided to withdraw life sustaining therapies?' is equal to 'Other' answer this question:***<br>What other information was used to decide on withdrawing life sustaining therapies?	
7.3.1.1	***If 'What was the cause of death?' is equal to 'Brain death' answer this question:***<br>How was brain death defined?<br>In case of a 'brain death procedure'; what were the criteria to support a brain death diagnosis? Clinically brain death, i.e. 1) GSC score of 3 w/o brainstem reflexes >24h post-ROC, 2) No sedation or neuromuscular blockade for at least 24h at time of neurologic examination using train of four and 3) Temperature > 32 degrees Celsius. Or brain death according to the 'whole brain death' concept according to Dutch law in order for DBD donation, i.e. 1) "prealabele voorwaarden": known cause, deathly and untreatable brain damage, no reversible causes, 2) drug-induced neurodepression is excluded, 3) neurologic examination is E1M1Vt, no stem reflexes and negative ice-water test, 4) EEG performed (and possibly TCD or CTA) and 5) apnea testing.	○ Clinically brain death according to described definitions<br>○ Brain death according to Dutch brain death protocol/law
7.6	Direct cause of arrest (Medical History and Cause of Arrest)	
7.7	Was the event etiology unresolved?<br>Select **Yes** if a clear cause of the arrest could not be found even after extensive (postmortem) investigations. If the arrest etiology remained unsure and multiple diagnosis were considered, also answer this question affirmative. Select **No** if a clear arrest etiology could be determined.	○ Yes<br>○ No
7.7.1	***If 'Was the event etiology unresolved?' is equal to 'Yes' answer this question:***<br>Investigations performed for unresolved events<br>Select all that apply. If the arrest etiology remained unresolved, what (postmortem) investigations were performed to determine an etiology/cause of death? Select "Unknown/Not documented" if not explicitly stated in thepatient's chart.	☐ Multidisciplinary staff meeting<br>☐ Blood culture testing<br>☐ Cardiac imaging (echocardiogram, CT/MRI)<br>☐ Detailed personal and family history taken<br>☐ Exercise test and/or provocative (medication) test<br>☐ Multiple ECG's and rhythm monitoring<br>☐ Toxicology and metabolic screening<br>☐ Cardiogenetic testing of patient<br>☐ Cardiogenetic testing of family<br>☐ Autopsy<br>☐ Postmortem MRI/CT<br>☐ Uknown/Not documented
7.8	Switch unresolved death: 1=Yes, 0=No	
7.8.1	***If 'Switch unresolved death: 1=Yes, 0=No' is equal to '1' answer this question:***<br>If a child with unresolved arrest etiology did not survive the event, how could it best be described?<br>**SUDS** - Unexplained sudden death occurring in an individual older than 1 year. **SIDS** - Unexplained sudden death occurring in an individual younger than 1 year with negative pathological and toxicological assessment **SADS** - Unexplained sudden death occurring in an individual older than 1 year with negative pathological and toxicological assessment. **SUDEP** - Sudden and unexpected, nontraumatic and nondrowning death of a person with epilepsy, without a toxicological or anatomical cause of death detected during the postmortem examination.	○ Sudden unexplained death syndrome<br>○ Sudden infant death syndrome<br>○ Sudden arrhythmic death syndrome<br>○ Sudden unexplained death in epilepsy
	**Comments**	
7.9	Outcome Comments<br>Do not lead entry with a "-" [dash] (will export as an error).	

8. Post-Cardiac Arrest Care and Neuroprognostication - General - Post-Cardiac Arrest Care

Number	Question	Answers
	**Complete this form ONLY if patient SURVIVED the cardiac arrest EVENT (i.e. achieved ROC!)**	
8.1	Index Arrest Date (Demographic and Patient Characteristics)<br>Enter date of the index arrest for this patient. The index arrest is the first cardiac arrest leading to this inpatient hospital stay (if it was an out of hospital arrest) or the first cardiac arrest of this current hospital stay (if this was an in-hospital event). (DD-MM-YYYY).	
8.2	Index Arrest Time (CPR Event Data)<br>Enter time of the index arrest for this patient. See definition for index arrest above. (HH:MM, military time).	
8.3	Did patient re-arrest this same PICU/hospital admission?<br>Yes- If patient have subsequent cardiac arrests after a period of sustained ROC.	◯ Yes<br>◯ No
8.3.1	***If 'Did patient re-arrest this same PICU/hospital admission?' is equal to 'Yes' answer this question:***<br>Date and time of re-arrest (1)<br>Enter the date and time of the subsequent re-arrest(s). (DD/MM/YYYY, HH:MM). There are up to two spots to enter date and times of subsequent cardiac arrests. If patient had more than 2 arrests, please make a note of that in the "Notes" section at the end of this form.	___ ___ ___ *(dd-mm-yyyy)*<br>___ : ___ *(hh:mm)*
8.3.2	***If 'Did patient re-arrest this same PICU/hospital admission?' is equal to 'Yes' answer this question:***<br>Total CPR duration: Total minutes of CPR for this event (min). If unsure, provide an educated estimate rounded UP to the nearest 5-minute interval.<br>Indicate total time of CCs from the first CC to the beginning of sustained ROSC (>20 minutes) or death. • If Yes was selected above, calculate total duration of CPR given during this resuscitation event. Should include both out of hospital and in hospital (if applicable) CPR time. Use direct observation, video monitoring, or CPR data to calculate the total duration of CPR. • If ROSC was attained for less than 20 minutes and chest compressions resumed, enter all epochs of chest compressions required during this event. • The event does not end until ROSC lasts >20 minutes. • If unsure, provide an educated estimate rounded UP to the nearest 5-minute interval.	______ minute(s)
8.3.3	***If 'Did patient re-arrest this same PICU/hospital admission?' is equal to 'Yes' answer this question:***<br>Date and time of re-arrest (2)<br>Enter the date and time of the subsequent re-arrest(s). (DD/MM/YYYY, HH:MM). There are up to two spots to enter date and times of subsequent cardiac arrests. If patient had more than 2 arrests, please make a note of that in the "Notes" section at the end of this form.	___ ___ ___ *(dd-mm-yyyy)*<br>___ : ___ *(hh:mm)*
8.3.4	***If 'Did patient re-arrest this same PICU/hospital admission?' is equal to 'Yes' answer this question:***<br>Total CPR duration: Total minutes of CPR for this event (min). If unsure, provide an educated estimate rounded UP to the nearest 5-minute interval.<br>Indicate total time of CCs from the first CC to the beginning of sustained ROSC (>20 minutes) or death. • If Yes was selected above, calculate total duration of CPR given during this resuscitation event. Should include both out of hospital and in hospital (if applicable) CPR time. Use direct observation, video monitoring, or CPR data to calculate the total duration of CPR. • If ROSC was attained for less than 20 minutes and chest compressions resumed, enter all epochs of chest compressions required during this event. • The event does not end until ROSC lasts >20 minutes. • If unsure, provide an educated estimate rounded UP to the nearest 5-minute interval.	______ minute(s)
8.4	Erasmus MC Sophia post-ROC protocol 1	
8.5	Erasmus MC Sophia post-ROC protocol 2	
	**Comments**	
8.6	Post-cardiac arrest care comments<br>Do not lead entry with a "-" [dash] (will export as an error).	

9. Post-Cardiac Arrest Care and Neuroprognostication - Airway/Breathing

Number	Question	Answers
9.1	Did patient go on invasive mechanical ventilation?<br>**Yes** - if patient was intubated and placed on invasive mechanical ventilation before, during or after resuscitation event.<br>**No** - if patient was never placed on invasive mechanical ventilation. Invasive mechanical ventilation is defined as the delivery of positive pressure to the lungs via an endotracheal or tracheostomy tube.	◯ Yes<br>◯ No
9.1.1	***If 'Did patient go on invasive mechanical ventilation?' is equal to 'Yes' answer this question:***<br>Mechanical ventilation start date and time<br>Start date and time of mechanical ventilation.	___ ___ ___ *(dd-mm-yyyy)*<br>___ : ___ *(hh:mm)*
9.1.2	***If 'Did patient go on invasive mechanical ventilation?' is equal to 'Yes' answer this question:***<br>Mechanical ventilation stop date and time<br>Date and time of mechanical ventilation treatment was stopped.	___ ___ ___ *(dd-mm-yyyy)*<br>___ : ___ *(hh:mm)*
9.2	Did patient receive a tracheal canulla?<br>**Yes** - if patient received a tracheal canule before, during or after resuscitation event and either weaned from the canule and was discharged without or was discharged with the tracheal canule in situ. **No** - if patient never received a tracheal canule.	◯ No<br>◯ Yes and discharged from the PICU without tracheal canule<br>◯ Yes and discharged from the PICU with tracheal canule
9.2.1	***If 'Did patient receive a tracheal canulla?' is not equal to 'No' answer this question:***<br>Why did the patient receive a tracheal canulla?<br>Select **Medical history** only if the main reason to place a tracheal cannula was an already existing condition. A specific condition could also be the main reason if it worsened due to the cardiac arrest event.	◯ To wean from mechanical ventilation<br>◯ Airway blockages<br>◯ To clean and remove secretions from the airway<br>◯ Medical history (congenital defects, neurologic impairments)
	**Comments**	
9.3	Post-cardiac arrest care comments<br>Do not lead entry with a "-" [dash] (will export as an error).	

10. Post-Cardiac Arrest Care and Neuroprognostication - Circulation

Number	Question	Answers
10.1	First (preferably arterial) blood gas after ROC<br>Are any blood samples containing pH / pCO2 / pO2 / Lactate values available within 12 hours after ROC? An **arterial** blood gas sample at any time point within 12 hours after ROC is the preferred source, then venous, then capillary. Enter not available if first blood gas is > 12 hours after ROC or time measurement of first blood gas is unknown/not documented.	○ Arterial<br>○ Venous<br>○ Capillairy<br>○ Not available
10.1.1	***If 'First (preferably arterial) blood gas after ROC' is not equal to 'Not available' answer this question:***<br>Date and time first blood gas after ROC	(dd-mm-yyyy)<br>: (hh:mm)
10.1.2	***If 'First (preferably arterial) blood gas after ROC' is not equal to 'Not available' answer this question:***<br>First blood gas pH after ROC<br>Whole number; x.yy	pH
10.1.3	***If 'First (preferably arterial) blood gas after ROC' is not equal to 'Not available' answer this question:***<br>First blood gas pCO2 after ROC<br>Whole number; x(x).yy	kPa
10.1.4	***If 'First (preferably arterial) blood gas after ROC' is not equal to 'Not available' answer this question:***<br>First blood gas pO2 after ROC<br>Whole number; x(x).yy	kPa
10.1.5	***If 'First (preferably arterial) blood gas after ROC' is not equal to 'Not available' answer this question:***<br>First blood gas lactate after ROC<br>x(x).yy	mmol/l
10.2	Did patient go on ECMO post-ROC?<br>**Yes** - if patient was cannulated to ECMO (extracorporeal membrane oxygenation) after resuscitation event. **No** - if patient was never placed on ECMO or prior to / during resuscitation event.	○ Yes<br>○ No
10.2.1	***If 'Did patient go on ECMO post-ROC?' is equal to 'Yes' answer this question:***<br>If placed on ECMO post-ROC, indicate type of ECMO<br>Indicate ECMO the type of pre-arrest or post-ROSC initiated ECMO.	○ Venoarterial (VA)<br>○ Venovenous (VV)
10.2.2	***If 'Did patient go on ECMO post-ROC?' is equal to 'Yes' answer this question:***<br>ECMO start date and time<br>Start date and time of ECMO treatment.	(dd-mm-yyyy)<br>: (hh:mm)
10.2.3	***If 'Did patient go on ECMO post-ROC?' is equal to 'Yes' answer this question:***<br>ECMO stop date and time<br>Date and time of ECMO treatment was stopped.	(dd-mm-yyyy)<br>: (hh:mm)
10.3	Did patient receive an Echocardiogram?<br>Yes- An ultrasound study was performed. Select only if patient received an ultrasound from time of ROC to 4 days post ROSC (within 96 hours time window after ROC).	○ Yes<br>○ No
10.3.1	***If 'Did patient receive an Echocardiogram?' is equal to 'Yes' answer this question:***<br>Date and time initiated<br>Enter the date and time initiated of the first ultrasound performed following sustained ROC. (DD-MM-YYYY, HH:MM).	(dd-mm-yyyy)<br>: (hh:mm)
10.3.2	***If 'Did patient receive an Echocardiogram?' is equal to 'Yes' answer this question:***<br>Echocardiam results<br>Please copy the full findings from the official cardiologist report of the cardiogram.	
10.4	Did patient go on renal replacement therapy?<br>During the ICU admission following ROC (or within the same admission for in-hospital ICU arrests) was there need for renal replacement therapy in the form of **Continuous Veno-Venous Hemofiltration (CVVH)** specifically (a temporary treatment for patients with acute renal failure who are unable to tolerate hemodialysis and are unstable).	○ Yes<br>○ No
10.4.1	***If 'Did patient go on renal replacement therapy?' is equal to 'Yes' answer this question:***<br>Why did patient go on renal replacement therapy?<br>Select the main reason for starting renal replacement therapy.	○ Acute kidney injury<br>○ Fluid overload<br>○ Other<br>○ Not documented/Unknown
10.4.1.1	***If 'Why did patient go on renal replacement therapy?' is equal to 'Other' answer this question:***<br>Why did patient go on renal replacement therapy?<br>Specify **Other** reason for starting renal replacement therapy.	
10.4.2	***If 'Did patient go on renal replacement therapy?' is equal to 'Yes' answer this question:***<br>CVVH start date and time<br>Start date and time of CVVH treatment.	(dd-mm-yyyy)<br>: (hh:mm)
10.4.3	***If 'Did patient go on renal replacement therapy?' is equal to 'Yes' answer this question:***<br>CVVH stop date and time<br>Date and time of CVVH treatment was stopped.	(dd-mm-yyyy)<br>: (hh:mm)
	**Comments**	
10.5	Post-cardiac arrest care comments<br>Do not lead entry with a "-" [dash] (will export as an error).	

11. Post-Cardiac Arrest Care and Neuroprognostication - Disabilities - examination

Number	Question	Answers
	**Serial Measurements**: From Time = 0 (time of ROC) until PICU discharge, select for each increment wether or not the monitoring values from patient's medical record will be entered.	
11.1	Time intervals<br>Select whether or not the neurological examination from patient's medical record will be entered for one or more increments. Please try to fill in all provided time increments.	○ Yes<br>○ No
11.2	Switch pcm_emc_hourly_measurements_yn 1, 0 or -1 (empty)	
11.1.1	***If 'Time intervals' is equal to 'Yes' answer this question:***<br>Neurological examination time point or intervals<br>Check each increment for which the neurological examination from patient's medical record will be entered.	☐ Directly post-ROC (IHCA) or upon PICU admission (OHCA), hour 0 - 1<br>☐ Hours 1 - 24<br>☐ Hours 24 - 78<br>☐ PICU discharge or day of death (if not captured in previous intervals)

12. Post-Cardiac Arrest Care and Neuroprognostication - Disabilities - examination t = 0 - 1 hour

Number	Question	Answers
	Step is not applicable. Check this increment in step Neurological Examination to enable this step.	
	Step is not applicable. Check this increment in step Neurological Examination to enable this step.	
12.1	Were any sedatives or muscle relaxants administered during or prior to this neurological examination?<br>**Yes** - if any sedative agent or muscle relaxant was administered during the neurological examination or if the neurological examination took place within the clearance time of this/these sedative(s). **No** - if no sedatives were administered or if according to the half time of the administered sedative, the agent was already cleared.	○ Yes<br>○ No
12.1.1	***If 'Were any sedatives or muscle relaxants administered during or prior to this neurological examination?' is equal to 'Yes' answer this question:***<br>What sedatives and/or muscle relaxants were adminstered?<br>Select all sedatives and/or muscle relaxants that were administered and had an effect on the patient's neurology during examination.	☐ Lorazapam<br>☐ Midazolam<br>☐ Propofol<br>☐ Clonidine/Dexmedetomidine<br>☐ Morfine<br>☐ Remifentanil<br>☐ Sufentanil<br>☐ Ketamine<br>☐ Haldol<br>☐ Epidural analgesia<br>☐ Succinylcholine<br>☐ Rocuronium
12.2	Glasgow Coma Scale	
12.3	GCS at ROC-IH/Adm-OOH Eye<br>Enter a value from 1 to 4. For in-hospital arrests, enter the Glasgow Coma Scale (GCS) immediately post ROC. For out-of-hospital arrests, enter the GCS for patients upon admission.	
12.4	GCS at ROC-IH/Adm-OOH Motor<br>Enter a value from 1 to 6. For in-hospital arrests, enter the Glasgow Coma Scale (GCS) immediately post ROC. For out-of-hospital arrests, enter the GCS for patients upon admission.	
12.5	GCS at ROC-IH/Adm-OOH Verbal<br>Enter a value from 1 to 5. Enter 1 for invasively ventilated patients. For in-hospital arrests, enter the Glasgow Coma Scale (GCS) immediately post ROC. For out-of-hospital arrests, enter the GCS for patients upon admission.	
12.6	GCS ROC Total 0 hours	
12.7	Pupillary reflex at ROSC-IH/Adm-OOH<br>Enter best pupillary reflex for this time increment. For in-hospital arrests, enter the pupillary reflex immediately post ROC. For out-of-hospital arrests, enter the pupillary reflex for patients upon PICU admission.	○ Present reflexes (bilaterally)<br>○ Absent reflex (unilaterally)<br>○ Absent reflexes (bilaterally)<br>○ Unknown/Not documented
12.8	Corneal reflex at ROSC-IH/Adm-OOH<br>Enter best corneal reflex for this time increment. For in-hospital arrests, enter the pupillary reflex immediately post ROC. For out-of-hospital arrests, enter the pupillary reflex for patients upon PICU admission.	○ Normal reflexes (bilaterally)<br>○ Absent reflex (unilaterally)<br>○ Absent reflexes (bilaterally)<br>○ Unknown/Not documented
12.9	Was the cough reflex present?<br>Enter the cough reflex for this time increment. For in-hospital arrests, enter the couch reflex immediately post ROC if available. For out-of-hospital arrests, enter the cough reflex for patients upon PICU admission.	○ Present<br>○ Absent<br>○ Unknown/Not documented
12.10	Was the oculocephalic reflex present?<br>Enter the oculocephalic reflex for this time increment. For in-hospital arrests, enter the oculocephalic reflex immediately post ROC if available. For out-of-hospital arrests, enter the oculocephalic reflex for patients upon PICU admission.	○ Present<br>○ Absent<br>○ Unknown/Not documented
12.11	Were there signs of spontaneous breathing/triggering?<br>Enter if there were signs of spontaneous breathing for this time increment. For in-hospital arrests, enter signs of spontaneous breathing immediately post ROC. For out-of-hospital arrests, enter signs of spontaneous breathing upon PICU admission.	○ Present<br>○ Absent<br>○ Unknown/Not documented
12.12	Where any of the following neurological signs present at this time?<br>**Nonepileptic myoclonus** - repetitive, generalized, focal or multifocal, motor myoclonic movements involving the face, limbs, or trunk that can occur at any time following CA. **Myoclonic status** - Clinical appearance of nearly continuous myoclonic jerking for at least 30 minutes. **Paroxysmal sympathetic hyperactivity** - PSH is defined as a clinical syndrome manifested by paroxysmal episodes of sympathetic activity that occur in patients with severe acute brain injury. Core clinical features include tachycardia, hypertension, tachypnea, hyperthermia, sweating, and/or increased muscle tone with possible dystonic posturing. **Not present** - only select this option if it is clearly states in the electronic patient file that abovementioned neurological signs were **not** present. Otherwise if not clearly states, select **Unknown/Not documented**.	☐ Nonepileptic myoclonus (< 30 minutes)<br>☐ Myoclonic status (> 30 minutes)<br>☐ Paroxysmal sympathetic hyperactivity<br>☐ Not present<br>☐ Uknown/Not documented
	**Comments**	
12.13	Post-cardiac arrest care comments<br>Do not lead entry with a "-" [dash] (will export as an error).	

13. Post-Cardiac Arrest Care and Neuroprognostication - Disabilities - examination t = 1 - 24 hours

Number	Question	Answers
	Step is not applicable. Check this increment in step Neurological Examination to enable this step.	
	Step is not applicable. Check this increment in step Neurological Examination to enable this step.	
13.1	Where any sedatives or muscle relaxants administerend during or prior to this neurological examination?<br>**Yes** - if any sedative agent and/or muscle relaxant was administered during the neurological examination or if the neurological examination took place within the clearance time of this/these sedative(s). **No** - if no sedatives were administered or if according to the half time of the administered sedative, the agent was already cleared.	○ Yes<br>○ No
13.1.1	***If 'Where any sedatives or muscle relaxants administerend during or prior to this neurological examination?' is equal to 'Yes' answer this question:***<br>What sedatives and/or muscle relaxants were adminstered?<br>Select all sedatives and/or muscle relaxants that were administered and had an effect on the patient's neurology during examination.	☐ Lorazapam<br>☐ Midazolam<br>☐ Propofol<br>☐ Clonidine/Dexmedetomidine<br>☐ Morfine<br>☐ Remifentanil<br>☐ Sufentanil<br>☐ Ketamine<br>☐ Haldol<br>☐ Epidural analgesia<br>☐ Succinylcholine<br>☐ Rocuronium
13.2	Glasgow Coma Scale	
13.3	GCS at 1 - 24 hours Eye<br>Enter a value from 1 to 4. Enter the best/highest GSC for this time increment.	
13.4	GCS at 1 - 24 hours Motor<br>Enter a value from 1 to 6. Enter the best/highest GSC for this time increment.	
13.5	GCS at 1 - 24 hours Verbal<br>Enter a value from 1 to 5. Enter 1 for invasively ventilated patients. Enter the best/highest GSC for this time increment.	
13.6	GCS ROSC Total 1 - 24 hours	
13.7	Pupillary reflex at 1 - 24 hours<br>Enter best pupillary reflex for this time increment.	○ Present reflexes (bilaterally)<br>○ Absent reflex (unilaterally)<br>○ Absent reflexes (bilaterally)<br>○ Unknown/Not documented
13.8	Corneal reflex at 1 - 24 hours<br>Enter best corneal reflex for this time increment.	○ Normal reflexes (bilaterally)<br>○ Absent reflex (unilaterally)<br>○ Absent reflexes (bilaterally)<br>○ Unknown/Not documented
13.9	Was the cough reflex present?<br>Enter the cough reflex for this time increment.	○ Present<br>○ Absent<br>○ Unknown/Not documented
13.10	Was the oculocephalic reflex present?<br>Enter the oculocephalic reflex for this time increment.	○ Present<br>○ Absent<br>○ Unknown/Not documented
13.11	Were there signs of spontaneous breathing/triggering?<br>Enter if there were signs of spontaneous breathing for this time increment.	○ Present<br>○ Absent<br>○ Unknown/Not documented
13.12	Where any of the following neurological signs present at this time?<br>**Nonepileptic myoclonus** - repetitive, generalized, focal or multifocal, motor myoclonic movements involving the face, limbs, or trunk that can occur at any time following CA. **Myoclonic status** - Clinical appearance of nearly continuous myoclonic jerking for at least 30 minutes. **Paroxysmal sympathetic hyperactivity** - PSH is defined as a clinical syndrome manifested by paroxysmal episodes of sympathetic activity that occur in patients with severe acute brain injury. Core clinical features include tachycardia, hypertension, tachypnea, hyperthermia, sweating, and/or increased muscle tone with possible dystonic posturing. **Not present** - only select this option if it is clearly states in the electronic patient file that abovementioned neurological signs were **not** present. Otherwise if not clearly states, select **Unknown/Not documented**.	☐ Nonepileptic myoclonus (< 30 minutes)<br>☐ Myoclonic status (> 30 minutes)<br>☐ Paroxysmal sympathetic hyperactivity<br>☐ Not present<br>☐ Uknown/Not documented
	**Comments**	
13.13	Post-cardiac arrest care comments<br>Do not lead entry with a "-" [dash] (will export as an error).	

14. Post-Cardiac Arrest Care and Neuroprognostication - Disabilities - examination t = 24 - 72 hours

Number	Question	Answers
	Step is not applicable. Check this increment in step Neurological Examination to enable this step.	
	Step is not applicable. Check this increment in step Neurological Examination to enable this step.	
14.1	Where any sedatives or muscle relaxants administerend during or prior to this neurological examination?<br>**Yes** - if any sedative agent or muscle relaxant was administered during the neurological examination or if the neurological examination took place within the clearance time of this/these sedative(s). **No** - if no sedatives were administered or if according to the half time of the administered sedative, the agent was already cleared.	○ Yes<br>○ No
14.1.1	***If 'Where any sedatives or muscle relaxants administerend during or prior to this neurological examination?' is equal to 'Yes' answer this question:***<br>What sedatives and/or muscle relaxants were adminstered?<br>Select all sedatives and/or muscle relaxants that were administered and had an effect on the patient's neurology during examination.	☐ Lorazapam<br>☐ Midazolam<br>☐ Propofol<br>☐ Clonidine/Dexmedetomidine<br>☐ Morfine<br>☐ Remifentanil<br>☐ Sufentanil<br>☐ Ketamine<br>☐ Haldol<br>☐ Epidural analgesia<br>☐ Succinylcholine<br>☐ Rocuronium
14.2	Glasgow Coma Scale	
14.3	GCS at 24 - 72 hours Eye<br>Enter a value from 1 to 4. Enter the best/highest GSC for this time increment.	
14.4	GCS at 24 - 72 hours Motor<br>Enter a value from 1 to 6. Enter the best/highest GSC for this time increment.	
14.5	GCS at 24 - 72 hours Verbal<br>Enter a value from 1 to 5. Enter 1 for invasively ventilated patients. Enter the best/highest GSC for this time increment.	
14.6	GCS ROSC Total 24 - 78 hours	
14.7	Pupillary reflex at 24 - 72 hours<br>Enter best pupillary reflex for this time increment.	○ Present reflexes (bilaterally)<br>○ Absent reflex (unilaterally)<br>○ Absent reflexes (bilaterally)<br>○ Unknown/Not documented
14.8	Corneal reflex at 24 - 72 hours<br>Enter best corneal reflex for this time increment.	○ Normal reflexes (bilaterally)<br>○ Absent reflex (unilaterally)<br>○ Absent reflexes (bilaterally)<br>○ Unknown/Not documented
14.9	Was the cough reflex present?<br>Enter the cough reflex for this time increment.	○ Present<br>○ Absent<br>○ Unknown/Not documented
14.10	Was the oculocephalic reflex present?<br>Enter the oculocephalic reflex for this time increment.	○ Present<br>○ Absent<br>○ Unknown/Not documented
14.11	Were there signs of spontaneous breathing/triggering?<br>Enter if there were signs of spontaneous breathing for this time increment.	○ Present<br>○ Absent<br>○ Unknown/Not documented
14.12	Where any of the following neurological signs present at this time?<br>**Nonepileptic myoclonus** - repetitive, generalized, focal or multifocal, motor myoclonic movements involving the face, limbs, or trunk that can occur at any time following CA. **Myoclonic status** - Clinical appearance of nearly continuous myoclonic jerking for at least 30 minutes. **Paroxysmal sympathetic hyperactivity** - PSH is defined as a clinical syndrome manifested by paroxysmal episodes of sympathetic activity that occur in patients with severe acute brain injury. Core clinical features include tachycardia, hypertension, tachypnea, hyperthermia, sweating, and/or increased muscle tone with possible dystonic posturing. **Not present** - only select this option if it is clearly states in the electronic patient file that abovementioned neurological signs were **not** present. Otherwise if not clearly states, select **Unknown/Not documented**.	☐ Nonepileptic myoclonus (< 30 minutes)<br>☐ Myoclonic status (> 30 minutes)<br>☐ Paroxysmal sympathetic hyperactivity<br>☐ Not present<br>☐ Uknown/Not documented
	**Comments**	
14.13	Post-cardiac arrest care comments<br>Do not lead entry with a "-" [dash] (will export as an error).	

15. Post-Cardiac Arrest Care and Neuroprognostication - Disabilites - examination t = PICU discharge

Number	Question	Answers
	Step is not applicable. Check this increment in step Neurological Examination to enable this step.	
	Step is not applicable. Check this increment in step Neurological Examination to enable this step.	
15.1	Where any sedatives or muscle relaxants administerend during or prior to this neurological examination?<br>**Yes** - if any sedative agent or muscle relaxant was administered during the neurological examination or if the neurological examination took place within the clearance time of this/these sedative(s). **No** - if no sedatives were administered or if according to the half time of the administered sedative, the agent was already cleared.	○ Yes<br>○ No
15.1.1	***If 'Where any sedatives or muscle relaxants administerend during or prior to this neurological examination?' is equal to 'Yes' answer this question:***<br>What sedatives and/or muscle relaxants were adminstered?<br>Select all sedatives and/or muscle relaxants that were administered and had an effect on the patient's neurology during examination.	☐ Lorazapam<br>☐ Midazolam<br>☐ Propofol<br>☐ Clonidine/Dexmedetomidine<br>☐ Morfine<br>☐ Remifentanil<br>☐ Sufentanil<br>☐ Ketamine<br>☐ Haldol<br>☐ Epidural analgesia<br>☐ Succinylcholine<br>☐ Rocuronium
15.2	Glasgow Coma Scale	
15.3	GCS at PICU discharge Eye<br>Enter a value from 1 to 4. Enter the GSC at PICU discharge.	______
15.4	GCS at PICU discharge Motor<br>Enter a value from 1 to 6. Enter the GSC at PICU discharge.	______
15.5	GCS at PICU discharge Verbal<br>Enter a value from 1 to 5. Enter 1 for invasively ventilated patients. Enter the GSC at PICU discharge.	______
15.6	GCS ROSC Total PICU Discharge	
15.7	Pupillary reflex at PICU discharge<br>Enter best pupillary reflex for this time increment.	○ Present reflexes (bilaterally)<br>○ Absent reflex (unilaterally)<br>○ Absent reflexes (bilaterally)<br>○ Unknown/Not documented
15.8	Corneal reflex at PICU discharge<br>Enter best corneal reflex for this time increment.	○ Normal reflexes (bilaterally)<br>○ Absent reflex (unilaterally)<br>○ Absent reflexes (bilaterally)<br>○ Unknown/Not documented
15.9	Was the cough reflex present?<br>Enter the cough reflex for this time increment.	○ Present<br>○ Absent<br>○ Unknown/Not documented
15.10	Was the oculocephalic reflex present?<br>Enter the oculocephalic reflex for this time increment.	○ Present<br>○ Absent<br>○ Unknown/Not documented
15.11	Were there signs of spontaneous breathing/triggering?<br>Enter if there were signs of spontaneous breathing for this time increment.	○ Present<br>○ Absent<br>○ Unknown/Not documented
15.12	Where any of the following neurological signs present at this time?<br>**Nonepileptic myoclonus** - repetitive, generalized, focal or multifocal, motor myoclonic movements involving the face, limbs, or trunk that can occur at any time following CA. **Myoclonic status** - Clinical appearance of nearly continuous myoclonic jerking for at least 30 minutes. **Paroxysmal sympathetic hyperactivity** - PSH is defined as a clinical syndrome manifested by paroxysmal episodes of sympathetic activity that occur in patients with severe acute brain injury. Core clinical features include tachycardia, hypertension, tachypnea, hyperthermia, sweating, and/or increased muscle tone with possible dystonic posturing. **Not present** - only select this option if it is clearly states in the electronic patient file that abovementioned neurological signs were **not** present. Otherwise if not clearly states, select **Unknown/Not documented**.	☐ Nonepileptic myoclonus (< 30 minutes)<br>☐ Myoclonic status (> 30 minutes)<br>☐ Paroxysmal sympathetic hyperactivity<br>☐ Not present<br>☐ Uknown/Not documented
	**Comments**	
15.13	Post-cardiac arrest care comments<br>Do not lead entry with a "-" [dash] (will export as an error).	______

16. Post-Cardiac Arrest Care and Neuroprognostication - Disabilities - neuromonitoring and imaging

Number	Question	Answers
16.1	Did patient receive EEG monitoring?<br>Yes - there was continuous or routine EEG monitoring from time of ROC up to 72 hours post ROC.	○ Yes<br>○ No
16.1.1	***If 'Did patient receive EEG monitoring?' is equal to 'Yes' answer this question:***<br>Indicate type of EEG monitoring<br>Select the type of EEG monitoring first performed from the time of sustained ROC until 24 hours post ROC.	○ Continuous<br>○ Routine<br>○ Other
16.1.1.1	***If 'Indicate type of EEG monitoring' is equal to 'Other' answer this question:***<br>Indicate type of EEG monitoring<br>Specify other type of EEG monitoring.	
16.1.2	***If 'Did patient receive EEG monitoring?' is equal to 'Yes' answer this question:***<br>Date and time initiated<br>Enter the date and time initiated of the first EEG following sustained ROC was initiated. (DD-MM-YYYY, HH:MM).	*(dd-mm-yyyy)*<br>: *(hh:mm)*
16.1.3	***If 'Did patient receive EEG monitoring?' is equal to 'Yes' answer this question:***<br>Date and time stopped<br>Enter the date and time stopped of the first EEG following sustained ROC was initiated. (DD-MM-YYYY, HH:MM).	*(dd-mm-yyyy)*<br>: *(hh:mm)*
16.2	Were there during this admission any signs of seizures?<br>Select all that apply. Signs of seizures could be observed clinically, clinically with fitting EEG patterns or subclinical based on EEG patterns only.	☐ No<br>☐ Clinically<br>☐ EEG
16.2.1	***If 'Were there during this admission any signs of seizures?' is equal to 'Clinically' answer this question:***<br>What type of clinical epileptiform activity was seen?<br>If clinical seizures were observed during admission what type of epileptiform activity onset was seen.	○ Focal onset<br>○ Generalised onset<br>○ Unknown onset
16.2.2	***If 'Were there during this admission any signs of seizures?' is equal to 'Clinically' answer this question:***<br>Was the epileptiform activity a status epilepticus?<br>**Status epilepticus** - A seizure that lasts longer than 5 minutes, or having more than 1 seizure within a 5 minutes period, without returning to a normal level of consciousness between episodes.	○ Yes<br>○ No
16.3	Were any anti-epileptics started and why?<br>Based on whether or not signs of epileptic activity were already present when anti-epileptic agents were administered.	○ No<br>○ Yes, prophylactically<br>○ Yes, for treatment
16.3.1	***If 'Were any anti-epileptics started and why?' is not equal to 'No' answer this question:***<br>What anti-epileptics were started?<br>Select all that apply. Only select drugs that were specifically started to prevent or treat epilepsy.	☐ Midazolam<br>☐ Propofol<br>☐ Ketamine<br>☐ Fenytoin<br>☐ Valproic acid<br>☐ Levetiracetam<br>☐ Carbamazepine<br>☐ Oxcarbazepine<br>☐ Lamotrigine<br>☐ Lacosamide<br>☐ Pentoparbital<br>☐ Phenobarbital
16.4	Did patient receive NIRS monitoring?<br>Yes - there was NIRS monitoring from time of ROC up to 72 hours post ROC.	○ Yes<br>○ No
16.4.1	***If 'Did patient receive NIRS monitoring?' is equal to 'Yes' answer this question:***<br>Date and time initiated<br>Enter the date and time initiated when NIRS monitoring was started following sustained ROC. (DD-MM-YYYY, HH:MM).	*(dd-mm-yyyy)*<br>: *(hh:mm)*
16.5	Did patient receive a cranial ultrasound?<br>Yes- if patient received cranial ultrasound at any time following sustained ROC. Includes from time of ROC until hospital discharge for this event.	○ Yes<br>○ No
16.5.1	***If 'Did patient receive a cranial ultrasound?' is equal to 'Yes' answer this question:***<br>Date and time initiated<br>Enter the date and time initiated of the first cranial ultrasound following sustained ROC was initiated. (DD-MM-YYYY, HH:MM).	*(dd-mm-yyyy)*<br>: *(hh:mm)*
16.5.2	***If 'Did patient receive a cranial ultrasound?' is equal to 'Yes' answer this question:***<br>What were the cranial ultrasound findings?<br>Report the findings of the cranial ultrasound preferably by copying the formal radiologist report.	
16.6	Did patient receive a brain CT?<br>Yes- if patient received brain CT at any time following sustained ROC. Includes from time of ROC until hospital discharge for this event.	○ Yes<br>○ No
16.6.1	***If 'Did patient receive a brain CT?' is equal to 'Yes' answer this question:***<br>Date and time initiated<br>Enter the date and time initiated of the first brain CT following sustained ROC was initiated. (DD-MM-YYYY, HH:MM).	*(dd-mm-yyyy)*<br>: *(hh:mm)*

16.6.2	***If 'Did patient receive a brain CT?' is equal to 'Yes' answer this question:***<br>What were the brain CT findings?<br>Report the findings of the cerebral CT preferably by copying the formal radiologist report.	
16.7	Did patient receive an brain MRI?<br>Yes- if patient received Cerebral MRI at any time following sustained ROC. Includes from time of ROC until hospital discharge for this event.	◯ Yes<br>◯ No
16.7.1	***If 'Did patient receive an brain MRI?' is equal to 'Yes' answer this question:***<br>Date and time initiated<br>Enter the date and time initiated of the first Cerebral MRI performed following sustained ROC. (DD-MM-YYYY, HH:MM).	*(dd-mm-yyyy)*<br>*(hh:mm)*
16.7.2	***If 'Did patient receive an brain MRI?' is equal to 'Yes' answer this question:***<br>What were the brain MRI findings?<br>Report the findings of the cerebral MRI preferably by copying the formal radiologist report.	
16.8	Was a SSEP performed?<br>Yes - if a SSEP (Somatosensory Evoked Potentials test) was performed at any time in the post-ROC period.	◯ Yes<br>◯ No
16.8.1	***If 'Was a SSEP performed?' is equal to 'Yes' answer this question:***<br>Date and time SSEP<br>Enter the date and time of the first SSEP following sustained ROC was initiated. (DD-MM-YYYY, HH:MM).	*(dd-mm-yyyy)*<br>*(hh:mm)*
16.8.2	***If 'Was a SSEP performed?' is equal to 'Yes' answer this question:***<br>What was the result of the SSEP?<br>Choose which option applies.	◯ Bilaterally - N20 absent - good quality for interpretation<br>◯ Unilaterally - N20 absent - good quality for interpretation<br>◯ SSEP failed (bad quality for interpretation, unable to perform measurement)<br>◯ Unknown/Not documented
16.9	Were any biomarkers used?<br>Biochemical markers from damaged neurons and glial cells can be measured quantitively in cerebrospinal fluid or blood and used as predictors of long-term neurological outcome in patients who remain comatose after cardiac arrest. Select whether one or more of these biomarkers were measured in the post-ROC period.	◯ Yes<br>◯ No
16.9.1	***If 'Were any biomarkers used?' is equal to 'Yes' answer this question:***<br>Which biomarkers were used?<br>Select all that apply.	☐ Neuron-specific enolase<br>☐ S100 calcium-binding protein B<br>☐ Plasminogen activator inhibitor-1<br>☐ Glial fibrillary acidic protein<br>☐ Ubiquitin carboxyl-terminal esterase L1<br>☐ Neurofilament light<br>☐ Tau
16.9.2	***If 'Were any biomarkers used?' is equal to 'Yes' answer this question:***<br>Date and time of the biomarker measurement<br>Enter the date and time the biomarker measurements were performed following sustained ROC was initiated. (DD-MM-YYYY, HH:MM).	*(dd-mm-yyyy)*<br>*(hh:mm)*
	**Comments**	
16.10	Post-cardiac arrest care comments<br>Do not lead entry with a "-" [dash] (will export as an error).	

17. Post-Cardiac Arrest Care and Neuroprognostication - Environment

Number	Question	Answers
17.1	Did patient receive therapeutic temperature management?(Any targeted temperature management post ROC. Includes actively keeping the patient normothermic or actively cooling the patient.)<br>Yes- if the patient is receiving any targeted temperature management post ROC. This includes actively keeping the patient normothermic or actively cooling the patient. This should be explicitly documented in the patient's chart in resuscitation and post resuscitation notes.	○ Yes<br>○ No
17.1.1	***If 'Did patient receive therapeutic temperature management?(Any targeted temperature management post ROC. Includes actively keeping the patient normothermic or actively cooling the patient.)' is equal to 'Yes' answer this question:***<br>Target temperature<br>If the patient received targeted temperature management, enter the target or goal temperature. (in Celsius). Enter a value for patients being kept normothermic as well.	[ ] Celcius
17.1.2	***If 'Did patient receive therapeutic temperature management?(Any targeted temperature management post ROC. Includes actively keeping the patient normothermic or actively cooling the patient.)' is equal to 'Yes' answer this question:***<br>How long was the patient in target temperature management for?<br>Enter the number of hours therapeutic temperature management was continued for.	[ ] Hours
17.2	Did the patient develop any fever during the first 72 hours after ROC?<br>Fever is defined by a core temperature >or=38.3 degrees C.	○ Yes<br>○ No
	**Comments**	
17.3	Post-cardiac arrest care comments<br>Do not lead entry with a "-" [dash] (will export as an error).	[ ]

18. Functional Outcome - Functional Outcome

Number	Question	Answers
	**Serial Measurements**: From pre-arrest to young adulthood, select for each increment wether or not the functional outcomes from patient's medical record will be entered.	
18.1	Time intervals<br>Select wether or not the neurological examination from patient's medical record will be entered for one or more increments.	○ Yes<br>○ No
18.2	Switch hms_emc_hourly_measurements_yn 1, 0 or -1 (empty)	
18.1.1	***If 'Time intervals' is equal to 'Yes' answer this question:***<br>Functional outcome time point<br>Check each increment for which the neurological examination from patient's medical record will be entered. These time points serve as a possible outline based on the Erasmus MC Sophia HINT (Hypoxic Ischemic NeuroTrauma) follow-up. For every time point a date can be entered so these time points can also be used as serial measurements independent of specific timing.	☐ Pre-Arrest<br>☐ Hospital Discharge<br>☐ 3 - 6 Months Post-Arrest<br>☐ 12 Months Post-Arrest<br>☐ 24 Months Post-Arrest<br>☐ At 5 years of age<br>☐ At 8 years of age<br>☐ At 12 years of age<br>☐ At 17 years of age
18.1.2	***If 'Time intervals' is equal to 'Yes' answer this question:***<br>Neuropsychological outcome time point<br>Check whether at one or more time points in the patients follow-up, neuropsychological tests have been performed.	○ At one or more time points, neuropsychological follow-up has been performed<br>○ Neuropsychological follow-up has not been performed
	**Neuropsychological follow-up**: If neuropsychological testing has been done, please enter performed tests in the neuropsychological follow-up section. Again time points serve as example but the exact test date can be entered.	

19. Functional Outcome - Pre-Arrest Functioning

Number	Question	Answers
	Step is not applicable. Check this increment in step Functional Outcome to enable this step.	
	Step is not applicable. Check this increment in step Functional Outcome to enable this step.	
19.1	Pediatric Cerebral Performance Category	
19.2	Pre-arrest PCPC<br>See image above.	○ Normal<br>○ Mild disability<br>○ Moderate disability<br>○ Severe Disability<br>○ Coma/Vegetative State<br>○ Brain death<br>○ Unable to determine (inadequate documentation)
19.3	Functional Status Scale	
19.4	Pre-arrest FSS Mental Status<br>See image above. Enter "0.00" if unknown or unable to determine.	
19.5	Pre-arrest FSS Sensory Functioning<br>See image above. Enter "0.00" if unknown or unable to determine.	
19.6	Pre-arrest FSS Communication<br>See image above. Enter "0.00" if unknown or unable to determine.	
19.7	Pre-arrest FSS Motor Functioning<br>See image above. Enter "0.00" if unknown or unable to determine.	
19.8	Pre-arrest FSS Feeding<br>See image above. Enter "0.00" if unknown or unable to determine.	
19.9	Pre-arrest FSS Respiratory Status<br>See image above. Enter "0.00" if unknown or unable to determine.	
19.10	Total pre-arrest FSS<br>Totals will be automatically calculated (use value recorded closest to time point).	
19.11	Pre-Arrest School Level<br>Select school level pre-arrest. Either attending regular school (elementary of high school), regular school with extra services or special needs school.	○ Regular elementary school<br>○ Elementary school with extra school services<br>○ Special needs school (elementary or high school level)<br>○ High school ("VMBO")<br>○ High school ("HAVO")<br>○ High school ("VWO")<br>○ Not applicable
	**Comments**	
19.12	Comments<br>**Do not lead entry with a "-" [dash] (will export as an error).**	

20. Functional Outcome - Hospital Discharge

Number	Question	Answers
	Step is not applicable. Check this increment in step Functional Outcome to enable this step.	
	Step is not applicable. Check this increment in step Functional Outcome to enable this step.	
20.1	Pediatric Cerebral Performance Category	
20.2	Hospital Discharge PCPC<br>See image above.	○ Normal<br>○ Mild disability<br>○ Moderate disability<br>○ Severe Disability<br>○ Coma/Vegetative State<br>○ Brain death<br>○ Unable to determine (inadequate documentation)
20.3	Functional Status Scale	
20.4	Hospital Discharge FSS Mental Status<br>See image above. Enter "0.00" if unknown or unable to determine.	
20.5	Hospital Discharge FSS Sensory Functioning<br>See image above. Enter "0.00" if unknown or unable to determine.	
20.6	Hospital Discharge FSS Communication<br>See image above. Enter "0.00" if unknown or unable to determine.	
20.7	Hospital Discharge FSS Motor Functioning<br>See image above. Enter "0.00" if unknown or unable to determine.	
20.8	Hospital Discharge FSS Feeding<br>See image above. Enter "0.00" if unknown or unable to determine.	
20.9	Hospital Discharge FSS Respiratory Status<br>See image above. Enter "0.00" if unknown or unable to determine.	
20.10	Total Hospital Discharge FSS<br>Totals will be automatically calculated (use value recorded closest to time point).	
	**Comments**	
20.11	Comments<br>**Do not lead entry with a "-" [dash] (will export as an error).**	
